# Revisiting thermoelectric transport properties through a band nonparabolicity factor

**DOI:** 10.1093/nsr/nwaf216

**Published:** 2025-05-31

**Authors:** Jianbo Zhu, Ming Liu, Xingyan Dong, Jingyu Li, Peng-Fei Liu, Xin Chen, Zihang Liu, Yongsheng Zhang, Fengkai Guo, Jiehe Sui

**Affiliations:** State Key Laboratory of Precision Welding & Joining of Materials and Structures, Harbin Institute of Technology, Harbin 150001, China; State Key Laboratory of Precision Welding & Joining of Materials and Structures, Harbin Institute of Technology, Harbin 150001, China; State Key Laboratory of Precision Welding & Joining of Materials and Structures, Harbin Institute of Technology, Harbin 150001, China; Institute of High Energy Physics, Chinese Academy of Sciences, Beijing 100049, China; Spallation Neutron Source Science Center, Dongguan 523803, China; Institute of High Energy Physics, Chinese Academy of Sciences, Beijing 100049, China; Spallation Neutron Source Science Center, Dongguan 523803, China; Advanced Research Institute of Multidisciplinary Sciences, Qufu Normal University, Qufu 273165, China; State Key Laboratory of Precision Welding & Joining of Materials and Structures, Harbin Institute of Technology, Harbin 150001, China; Advanced Research Institute of Multidisciplinary Sciences, Qufu Normal University, Qufu 273165, China; State Key Laboratory of Precision Welding & Joining of Materials and Structures, Harbin Institute of Technology, Harbin 150001, China; State Key Laboratory of Precision Welding & Joining of Materials and Structures, Harbin Institute of Technology, Harbin 150001, China

**Keywords:** band structures, thermoelectric material, nonparabolicity, Lorenz number

## Abstract

Thermoelectric materials are advanced functional semiconductors for the forthcoming era of energy conversion. The development in this field critically depends on the understanding of their band structures. However, many analyses rely on the parabolic band model that oversimplifies the realistic electron behavior. Such simplification leads to significant deviations in the predicted behavior of electrical transport properties due to the non-local characteristics of the Seebeck coefficient and the Lorenz number. This study introduces a nonparabolicity factor *ξ*, which quantitatively measures the deviations from parabolic dispersion in semiconductor band structures and can also directly predict the thermoelectric performance change induced by the band nonparabolicity. Notably, our results reveal that the influence of band nonparabolicity is significant when estimating the Lorenz number. We have formulated a universal *ξ* modified solution for the Lorenz number, which can effectively correct the non-physical lattice thermal conductivity derived from the typical parabolic band model in various representative thermoelectric semiconductors, establishing a basis for further insights into the underlying mechanisms of electrical and thermal transport.

## Teaser

A nonparabolicity factor is introduced to resolve key inaccuracies in thermoelectric transport property evaluations, particularly improving predictions of lattice thermal conductivity in semiconductors.

## INTRODUCTION

The band structure is perhaps the most important characteristic for understanding and engineering various semiconductor devices, including categories such as photovoltaic [1], optoelectronic [2], piezoelectric [3] and thermoelectric [4] applications. In the field of thermoelectric materials, a category that has attracted considerable attention for their remarkable ability to directly harvest energy from waste heat [5,6], band engineering is recognized as a highly effective strategy to enhance performance [7]. A variety of strategies rooted in band engineering, such as band convergence [8,9], resonance energy level design [10,11] and band sharpening [12,13], have been proven successful in improving thermoelectric performance. Therefore, uncovering the connections between band structures and thermoelectric properties is pivotal in the design of materials, enabling the precise engineering of high-performance thermoelectric solutions.

**Figure 1.**
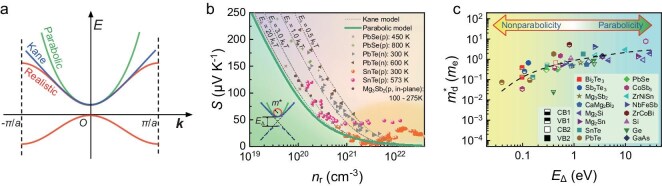
Comparison of band structure models and their impact on thermoelectric properties. (a) Diagrams of band structure models, including the parabolic model, Kane model and realistic band under the period boundary condition. (b) Modified Pisarenko plot under the parabolic and Kane band model. Solid symbols represent experimental results, including n-type PbTe [18], p-type PbSe [19], SnTe [20] and Mg_3_Sb_2_ [21]. The shaded region at large *n*_r_ indicates band convergence of SnTe materials. (c) Fitted density-of-state effective mass and parameter *E*_Δ_ of various semiconductors. The dashed line is guide for eyes.

Generally, the electronic transport behavior of semiconductor materials is predominantly influenced by the characteristics near the band extremities. A parabolic dispersion relation, as illustrated in Fig. 1a, represented as *E* = ${\hbar}$^2^*k*^2^/2m*, where m* denotes the effective mass of a carrier (the electron or hole), is widely employed to model the carrier transport in bulk semiconductors. On the basis of a direct analytic dispersion relation, also referred to as the parabolic band (PB) model [14], varieties of thermoelectric transport properties, including electrical conductivity (*σ*), Seebeck coefficient (*S*), Lorenz number (*L*) and electronic thermal conductivity (*κ*_e_ = *LσT*), can be established by using the semi-classical Boltzmann transport theory [15,16]. Notably, it is feasible to conversely reconstructed key band characteristics of the material from experimental data within the PB model, as highlighted in our previous work [17]. In the research and exploration of thermoelectric materials, the PB model plays a vital role, effectively bridging the divide between theoretical band structures and practical thermoelectric properties.

However, significant inconsistency emerges when attempting to align those key band characteristics reconstructed from experimental results [18–21] using the PB model to finely calculate the band structure. Under the PB model, the Pisarenko plot, which plots the Seebeck coefficient against the carrier concentration (*n*), is solely dependent on the density-of-states effective mass (*m*_d_*) and temperature (*T*). Moreover, we can define the reduced carrier concentration as *n*_r_ = *n*·(*m*_d_*/*m*_e_·*T*/*T*_r_)^−3/2^, where *m*_e_ is the mass of the free electron and *T*_r_, set at 300 K in general, is used for dimensionless calculations. According to the PB model [14], the *S*–*n*_r_ plot should adhere to a definitive material-independent curve, as illustrated in Fig. 1b. However, when the calculated *m*_d_* and experimental (*S, n*) results are collected to gain (*S, n*_r_) points (n-type PbTe [18], p-type PbSe [19], SnTe [20] and Mg_3_Sb_2_ [21]), a significant deviation from predictions of the PB model can be observed, particularly in the lightly doped materials. Notably, these deviations cannot be attributed solely to band convergence or the resonance energy level, which is typically observed in heavily doped materials, thereby suggesting the need for an alternative explanation.

Due to the constraints imposed by periodic boundary conditions and complex interatomic interactions, the PB model often inadequately describes the band dispersions of semiconductor materials, particularly those with narrow band gaps. In the context of interatomic interactions, it is nearly impossible to keep a rapid increase consistent with the trend of *k*^2^ as *k* moves away from the extrema of the energy bands, as schematically represented in Fig. 1a (see Fig. S1 and Note S1 for a discussion on modeling the central equation). A widely embraced refinement, known as the Kane model [22,23], defined by the equation *E*(1 + *E*/*E*_Δ_) = ${\hbar}$^2^*k*^2^/2m*, incorporates the parameter *E*_Δ_ to more accurately describe the actual band structure (Fig. S2 and Note S2). Theoretically, as *E*_Δ_ approaches infinity (rendering *E*/*E*_Δ_ negligible), the Kane model converges to the classical PB model. In contrast, as *E*_Δ_ approaches zero and *E*/*E*_Δ_ increases substantially, the model exhibits increased deviations, indicating strong nonparabolicity and a significant divergence from typical free-electron behavior. The fitted *m*_d_* and *E*_Δ_ of some typical thermoelectric materials and classical functional semiconductors are presented in Fig. 1c (see details in Figs S3 and S4). As illustrated in Fig. 1b, adjusting *E*_Δ_ allows the predicted *S*–*n_r_* plots to align more closely with the experimental results. Beyond the Kane band model, advancements in band structure calculations have revealed increasingly intricate features, such as warping bands [24], quasi-1D pudding-mold-type bands [25], Rashba-split bands [26] and non-ellipsoidal bands [27]. While such studies provide deeper insights into the thermoelectric properties of specific materials, they often rely on material-specific, computationally intensive methods, limiting their ability to offer broader guidance for thermoelectric design across diverse systems [28,29]. This trend highlights the challenge of translating complex electronic characteristics into universally applicable models [30]. Addressing this gap, the analytical tractability of the Kane model offers a robust alternative to the classical PB approximation. By building on its clear physical foundation and extending its scope, this approach seeks to establish a transparent framework capable of supporting both experimental investigations and theoretical exploration in a wide range of thermoelectric materials.

In this work, we revisited the semi-classical Boltzmann transport theory to elucidate the influence of band nonparabolicity on thermoelectric properties. We then demonstrated that *S* and *L* exhibit strong non-local characteristics in the energy dimension, deviating from the common *σ* that depends on the local band structure near the Fermi level. This suggests that band dispersion deep within the valence or conduction band also influences the properties of carriers in intrinsic semiconductors. Additionally, we introduce a parameter *ξ*, referred to as the nonparabolicity factor, to characterize the band nonparabolicity and quantify its impact on thermoelectric properties. In the Kane band model, *ξ* is constrained to a range of 0–1, serving as a direct quantification of the nonparabolicity of the band structure. Finally, we illustrate our findings by demonstrating how the non-PB model addresses the non-physical lattice thermal conductivity behavior observed in beryllium-doped SnTe samples and several typical thermoelectric semiconductors.

## RESULTS

### Band structure and nonparabolicity illustration

**Figure 2.**
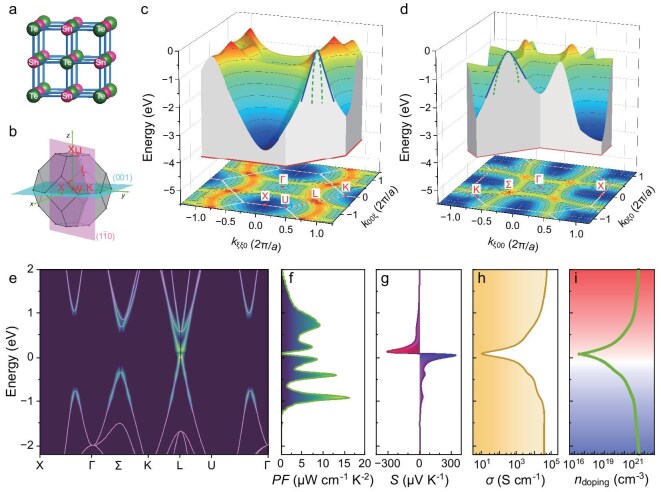
Structural and electronic properties of SnTe. (a) Crystal structure and (b) the first Brillouin zone of SnTe. (c) and (d) 3D valence band structures of SnTe on (${\bar{110}}$) and (001) planes in reciprocal space, respectively. (e) Band structure along the high symmetry path and *PF* dispersion. (f)–(i) *PF*, Seebeck coefficient, conductivity and carrier concentration with the Fermi level, respectively.

Taking the band structure of SnTe as an example, we visually illustrate the concept of band nonparabolicity. As depicted in Fig. 2a, SnTe has a cubic rock-salt structure [31], with its first Brillouin zone shown in Fig. 2b. It exhibits a prominent double valence band structure [32,33], with the valence band maximum located at the *L* point and a secondary maximum at the *Σ* point (on the *Γ*–*K* pathway). Figure 2c and d illustrates the 3D band structures on the *k*-planes that contain these valence band extrema. The shape of the 3D structures clearly indicates that, away from the band extrema, the energy varies almost linearly with the wavevector. Thus, regardless of the adjustments of m*, the parabolic curve (green dashed lines) produced by the quadratic PB model remains misaligned with the actual band structure, and is only valid within a limited energy range (<0.1 eV for the *L* valley). Meanwhile, the Kane curve (blue solid lines) closely matches the actual band structure up to an energy range of ∼1 eV when an appropriate *E*_Δ_ (0.16 eV for *L* valley and 0.51 eV for *Σ* valley) is employed. For thermoelectric materials, the electrical transport performance is commonly evaluated by the power factor (*PF* = *σS*^2^). The calculated power factor dispersion *PF*_s_ (see ‘Materials and methods’ section in the online Supplementary file for more details) in the reciprocal space for SnTe is shown in Fig. 2e, with additional thermoelectric properties depicted in Fig. 2f–i. It is evident that, although the regions near the band extrema play a significant role in determining the *PF*, the linearly dispersing regions also make notable contributions. Therefore, these linear regions in the band structure are also crucial factors to consider when evaluating thermoelectric performance.

### Boltzmann transport theory and nonparabolicity influence

**Figure 3.**
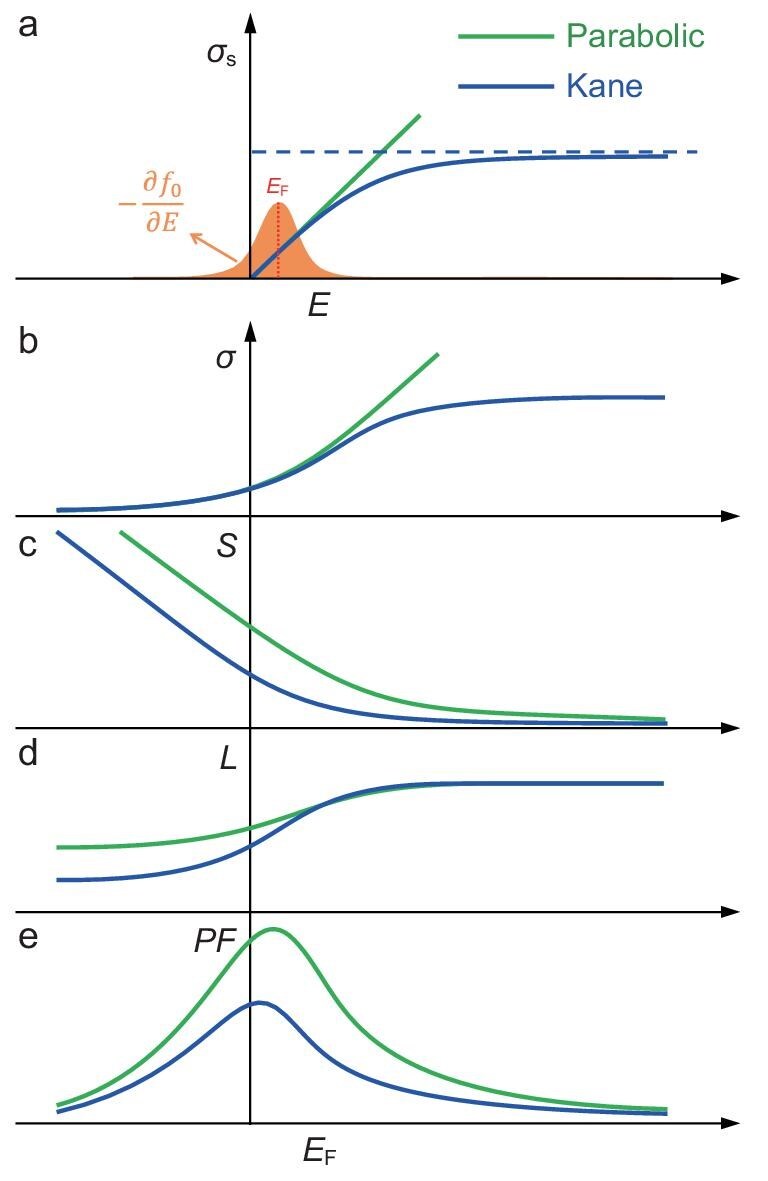
Schematic representation of thermoelectric transport properties under parabolic and Kane band models. (a) Transport distribution function. (b) Conductivity. (c) Seebeck coefficient. (d) Lorenz number. (e) *PF*.

To elucidate the relationship between the carrier dispersion relations and the thermoelectric transport properties, it is essential to revisit the Boltzmann transport theory. Under the relaxation time approximation, the transport distribution function *σ*_s_(*E*) is defined as in Equation (1):


(1) \begin{eqnarray*}
{\sigma }_s\left( E \right) = \frac{1}{3}{q}^2\tau v_g^2g\left( E \right),
\end{eqnarray*}

where *q* is the carrier charge, *τ* is the relaxation time, *v*_g_ is the group velocity and *g*(*E*) is the density-of-states of the carrier. It is generally assumed that the carrier scattering probability (*τ*⁻¹) is proportional to *g*(*E*), particularly when acoustic phonon scattering dominates (see Fig. S5). Consequently, *σ*_s_(*E*) is proportional to *v*_g_² and, under the PB model, it is also proportional to *E*. In the context of the Kane dispersion relation, assuming an identical *m*^∗^, *σ*_s_(*E*) near the band extremum behaves similarly to that of the PB model. However, as *E* increases, the Kane dispersion gradually transitions from a quadratic to a linear dependence, resulting in a constant *v*_g_ and a flat *σ*_s_(*E*), as depicted in Fig. 3a. Based on *σ*_s_(*E*), thermoelectric properties *σ, S* and *L* can be expressed by using Equations (2)–(4):


(2) \begin{eqnarray*}
\sigma = \mathop \int \nolimits_{ - \infty }^{ + \infty } {\sigma }_s\left( E \right) \cdot \left( { - \frac{{\partial {f}_0}}{{\partial E}}} \right)dE,
\end{eqnarray*}

(3) \begin{eqnarray*}
S = \frac{{{k}_B}}{q}\frac{{\mathop \smallint \nolimits_{ - \infty }^{ + \infty } {\sigma }_s\left( E \right) \cdot \left( {\frac{{E - {E}_F}}{{{k}_BT}}} \right) \cdot \left( { - \frac{{\partial {f}_0}}{{\partial E}}} \right)dE}}{{\mathop \smallint \nolimits_{ - \infty }^{ + \infty } {\sigma }_s\left( E \right) \cdot \left( { - \frac{{\partial {f}_0}}{{\partial E}}} \right)dE}},
\end{eqnarray*}

(4) \begin{eqnarray*}
L = {\left( {\frac{{{k}_B}}{q}} \right)}^2\frac{{\mathop \smallint \nolimits_{ - \infty }^{ + \infty } {\sigma }_s\left( E \right) \cdot {{\left( {\frac{{E - {E}_F}}{{{k}_BT}}} \right)}}^2 \cdot \left( { - \frac{{\partial {f}_0}}{{\partial E}}} \right)dE}}{{\mathop \smallint \nolimits_{ - \infty }^{ + \infty } {\sigma }_s\left( E \right) \cdot \left( { - \frac{{\partial {f}_0}}{{\partial E}}} \right)dE}} - {S}^2,
\end{eqnarray*}

where *k*_B_ is the Boltzmann constant, *E*_F_ is the Fermi level and *f*_0_ is the Fermi–Dirac distribution. The term –∂*f*_0_/∂*E* represents a Gaussian-like unimodal distribution function centered on *E*_F_, as shown in Fig. 3a. According to Equation (2), *σ* is the cumulative result of *σ*_s_(*E*) weighted by –∂*f*_0_/∂*E*, essentially representing the localized average of *σ*_s_(*E*) across the energy range near *E*_F_, as illustrated in Fig. 3b. Therefore, *σ* is primarily influenced by the band structure near the Fermi level, reflecting its local characteristics. For *S* and *L*, these quantities are not simply the cumulative results of weighted *σ*_s_(*E*), but rather the ratios of two cumulative integrals, as described by Equations (3) and (4). As *E*_F_ decreases into the bandgap (*E*_F_ << 0), reaching the non-degenerate semiconductor regime, –∂*f*_0_/∂*E* can be simplified to exp[(*E*_F_ – *E*)/(*k*_B_*T*)]. Consequently, these integrals tend to approach 0/0-type indeterminate forms. This complexity means that *S* and *L* cannot be described by the local characteristic observed in *σ*, leading to more complex behavior. As illustrated in Fig. 3c and d, *S* and *L* are similar for parabolic and Kane dispersion relations when *E*_F_ >> 0, despite significant differences in the dispersion relations. In contrast, both *S* and *L* differ greatly between parabolic and Kane dispersion relations when *E*_F_ << 0, when only rare carriers near the band extremum dominate the transport behavior, exhibiting pronounced non-local characteristics. These anomalies suggest that, even in semiconductors with very low doping levels, differences in band structure deep within the band can significantly affect the thermoelectric properties of the material. Hence, deviations from a parabolic dispersion can have substantial effects on the thermoelectric performance of the material.

### Band nonparabolicity factor and thermoelectric performance

**Figure 4.**
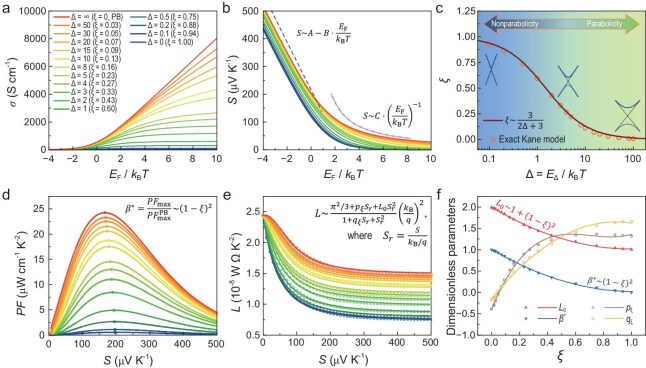
Thermoelectric properties of Kane model. (a) Conductivity and (b) Seebeck coefficient plots under varying band nonparabolicity. (c) Relationship between nonparabolicity factor and parameter Δ. (d) *PF* and (e) Lorenz number plots under varying band nonparabolicity. (f) Dependence of key dimensionless parameters on the nonparabolicity factor. Symbols in (c), (e) and (f) indicate results strictly solved from the Kane model, while the solid lines are fitting curves.

In the Kane model, the parameter Δ = *E*_Δ_/*k*_B_*T* acts as a knob to adjust the degree of deviation from the parabolic dispersion relation. Theoretically, when Δ approaches infinity, the Kane model degenerates to the PB model. As Δ decreases from infinity to zero, it signifies a transition of the band dispersion from a standard PB to a Dirac-like linear dispersion relation (*E* ∝ ${\hbar}$*k*). The *E*_F_ dependence of *σ* and *S* with varying Δ is illustrated in Fig. 4a and b, respectively. As the deviation from the parabolic relation increases, both of them degrade, indicating that the deviation from the parabolic dispersion negatively impacts the *PF*, thereby posing a challenge to achieving high thermoelectric performance. Quantifying the impact of such band nonparabolicity on thermoelectric performance is therefore essential. However, directly using Δ is practically inconvenient due to its semi-infinite nature. As is evident from Fig. 4b, in the region in which *E*_F_ << 0, *S* increases linearly with decreasing *E*_F_, regardless of the value of Δ, as described by *S* = A – B·*E*_F_/*k*_B_*T*. Here, the coefficient B is a material-independent constant (equal to *k*_B_/*q*), while A is determined by the dispersion relation and decreases monotonically with increasing Δ. Consequently, a dimensionless parameter *ξ* is introduced to quantify the band nonparabolicity, which is defined as Equation (5):


(5) \begin{eqnarray*}
\xi = \mathop {\lim }\limits_{{E}_{\mathrm{F}}\rightarrow - \infty } \frac{{{S}^{PB}\left( {{E}_F;T} \right) - S\left( {{E}_F;T} \right)}}{{{k}_B/q}},
\end{eqnarray*}

where *S*^PB^(*E*_F_; *T*) is the Seebeck coefficient under the PB model. When *E*_F_ is much smaller than 0, it reduces to a linear relationship, with the corresponding intercept A equal to 2·*k*_B_/*q* (≈ 172 μV K^−1^). The parameter *ξ* effectively measures the deviation of a band structure from the PB model. A vanishing *ξ*, occurring as Δ approaches infinity in the Kane model, indicates that the band structure is equivalent to the PB model in predicting thermoelectric properties. Conversely, an increasing *ξ* indicates a growing deviation from the PB model. Ultimately, when the dispersion relation degenerates into a completely linear form in the Kane model, i.e. when Δ is zero, *ξ* ≡ 1 (see Note S3). Thus, *ξ* always ranges from 0 to 1 within the Kane model, serving as an effective normalized quantitative parameter to describe band nonparabolicity.

Due to the bounded nature of the parameter *ξ*, a set of feasible expressions can be developed by using polynomial fitting or Pade approximation to link thermoelectric properties to it. Primarily, a fitted expression is established to relate *ξ* and Δ by *ξ* ≈ 3/(2Δ+3), as shown in Fig. 4c, which facilitates direct conversion between them. Subsequently, the impact of band nonparabolicity on *PF* can be examined from the perspective of *ξ*. Within the PB model framework, *PF* invariably reaches its maximum (*PF*_max_) when *S* is ∼167 μV K^−1^ [34] and *PF*_max_ = C·*μ*_0_(*m*_d_*/*m*_e_·*T*/*T*_r_)^3/2^ = C·*μ*_WT_, where *μ*_0_ represents the intrinsic mobility and C is a material-independent constant [17]. Taking *μ*_WT_ = 200 cm^2^ V^−1^ s^−1^, Fig. 4d shows the dependence of *PF* on *S* under varying degrees of nonparabolicity. As *ξ* increases, the *PF*_max_ of the material gradually decreases, while the corresponding *S* value increases to ∼200 μV K^−1^. This provides a more precise guideline for doping engineering to maximize *PF*, especially when experimental data on the carrier concentration are absent. Based on polynomial fitting, the ratio of the *PF*_max_ for a generic band to that of a standard PB model satisfies Equation (6):


(6) \begin{eqnarray*}
{\beta }^* = \frac{{P{F}_{max}}}{{PF_{max}^{PB}}} \approx {\left( {1 - \xi } \right)}^2.
\end{eqnarray*}

The deviation between the precisely calculated β* values and those obtained from the polynomial calculation is <2%, as shown in Fig. 4f. It should be noted that Equation (6) is derived by varying *E*_Δ_ to manipulate *ξ* in isolation while keeping m* unchanged, primarily for simplifying the analysis and isolating variables. However, in practice, manipulating *E*_Δ_ in isolation while maintaining m* unchanged is uncommon, as adjustments to *E*_Δ_ often lead to simultaneous changes in m*. When *E*_Δ_ and m* change synergistically, the relationship between *PF*_max_ and *ξ* may deviate from the monotonic decrease predicted by Equation (6). In Note S4, we explore a nonparabolicity adjustment pathway that maintains the ratio between *E*_Δ_ and m* (see example for Ref. [35]), demonstrating that *PF*_max_ can be enhanced as *ξ* increases under specific conditions.

The estimation of *L* is of great importance in thermoelectric experiments [36]. Typically, *L* is derived from the measured *S* and subsequently used to calculate *κ*_e_ via the Wiedemann–Franz law. This approach also allows estimation of the lattice thermal conductivity (*κ*_L_) by subtracting *κ*_e_ from the total thermal conductivity (*κ*), i.e. *κ*_L_ = *κ* – *κ*_e_. Theoretically, *L* can be expressed as *L*_r_·(*k*_B_/*q*)², where *L*_r_ is a dimensionless factor. In the PB model, *L*_r_ depends solely on *S* and decreases from π^2^/3 (typical of metallic systems) to 2 (typical of intrinsic semiconductors) as *S* increases [37]. However, as the band becomes more nonparabolic, in the framework of the Kane model, *L* follows a similar trend but exhibits noticeable numerical differences, as shown in Fig. 4e. By employing the mathematical concept of Pade approximation, a *L*–*S* relation with the correction factor *ξ* is analytically formulated, as expressed in Equation (7):


(7) \begin{eqnarray*}
L = \frac{{{\pi }^2/3 + {p}_\xi {S}_r + {L}_0S_r^2}}{{1 + {q}_\xi {S}_r + S_r^2}}{\left( {\frac{{{k}_B}}{q}} \right)}^2,
\end{eqnarray*}

where *S*_r_ = *S*/(*k*_B_/*q*) and *L*_0_, *p_ξ_* and *q_ξ_* are parameters that depend only on *ξ* and can be determined through Equations (8)–(10):


(8) \begin{eqnarray*}
{L}_0 = 2 - 2\xi + {\xi }^2,
\end{eqnarray*}

(9) \begin{eqnarray*}
{p}_\xi = - 0.33 + 6.90\xi - 7.78{\xi }^2 + 2.75{\xi }^3,
\end{eqnarray*}

(10) \begin{eqnarray*}
{q}_\xi = - 0.14 + 4.08\xi - 2.13{\xi }^2.
\end{eqnarray*}

The analytical *L*–*S* relation determined by using Equations (8)–(10) deviates by <3% from the exact numerical solutions (Fig. S10), providing a quick estimation method.

According to Equation (7), when *S* equals 0, *L*_r_ accurately reaches π^2^/3 and is not influenced by the band nonparabolicity. As *S* approaches infinity, however, *L*_r_ converges to *L*_0_, which is determined by the extent of the band nonparabolicity. Equation (8) shows that, when *ξ* is 0, *L*_0_ = 2, in line with the predictions from the PB model. When *ξ* reaches 1, *L*_0_ decreases to 1, which indicates significant nonparabolicity on the band structure, adhering to the simple PB model could introduce ≤50% deviation in the *L* estimation. To ensure accurate modeling over both finite and asymptotic behaviors, Pade approximations were employed in the derivation of these equations, as previously introduced. Unlike polynomial fitting methods, which often exhibit oscillatory behavior at infinity, Pade approximations provide a smooth transition between bounded interaction regions and asymptotic limits. This approach balances computational simplicity with physical reliability. It is crucial to highlight that, although band nonparabolicity appears in the deeper regions of the band, the significant disparities in *L* are primarily observed in lightly doped intrinsic semiconductors, further illustrating its non-local characteristics.

### Application of band nonparabolicity factor

**Figure 5.**
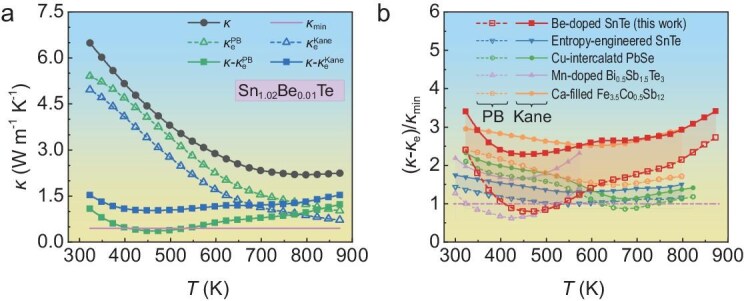
Thermal conductivities of Be-doped SnTe and typical thermoelectric materials. (a) Total thermal conductivity, electronic thermal conductivity and derived lattice thermal conductivity of Sn_1.02_Be_0.01_Te. (b) Ratio of the derived lattice thermal conductivity to the Debye–Cahill limit (*κ*_min_) for various thermoelectric materials, including entropy-engineered SnTe [41], Cu-intercalated PbSe [42], Mn-doped Bi_0.5_Sb_1.5_Te_3_ [40] and Ca-filled Fe_3.5_Co_0.5_Sb_12_ [43].

Back to SnTe, we synthesized polycrystalline SnTe samples with trace amounts (∼1 at.%) of Be substituting Sn sites. When subtracting *κ*_e_ calculated by using the *L* derived from the PB model, as shown in Fig. 5a, it is observed that the derived *κ*_L_ (0.35 W m^−1^ K^−1^ at 448 K) fell below the Debye–Cahill limit (*κ*_min_, 0.45 W m^−1^ K^−1^), which corresponds to a phonon mean free path that is half the wavelength and is often referred to as the amorphous limit of lattice thermal conductivity [38,39]. It is evident that a straightforward point defect scattering mechanism in Be-doped SnTe is inadequate to account for such intense phonon scattering. As discussed, the anomalous non-physical behavior observed in materials such as SnTe, which exhibits strong band nonparabolicity, can be attributed to the fact that the PB model tends to overestimate *L*, thereby significantly underestimating *κ*_L_. However, when the effects of band nonparabolicity are considered, with the temperature-dependent parameter *ξ* calculated by using the fitted value of *E*_Δ_ (0.18 eV), the derived *κ*_L_ of the material lies above the theoretical limit *κ*_min_, yielding a more physically reasonable result. In fact, the emergence of the second valence band also causes changes in *L* (see Fig. S8), necessitating more complex calculations that must be considered in heavily doped samples. However, Equations (7)–(10) provide an efficient way to estimate *L*, offering broader applicability compared with the conventional PB model.

Taking *κ*_min_ as a reference, Fig. 5b illustrates the derived *κ*_L_ for various thermoelectric materials. In the case of the typical narrow-bandgap thermoelectric material (Bi, Sb)_2_Te_3_ doped with Mn [40], the PB-derived *κ*_L_ unexpectedly fell to ∼40% lower than *κ*_min_. Such an unreasonable result can be addressed by incorporating band nonparabolicity, resulting in a derived *κ*_L_ that is not lower than *κ*_min_. Additionally, for entropy-engineered SnTe with significant lattice distortion and strong strain fluctuations [41], the derived *κ*_L_ remains superior to that of simple Be-doped samples at ∼450 K if the standard PB model is arbitrarily applied. Upon including band nonparabolicity, the derived *κ*_L_ of the entropy-engineered SnTe drops to below that of Be-doped samples, which is consistent with primary scientific principles. It is also important to note that the influence of nonparabolicity is not uniform across different materials [42,43], highlighting the necessity for our quantitative evaluation framework for *L*. Therefore, the *ξ*-modified solution for *L* provides a rapid and effective estimation for both electronic and lattice thermal conductivity, which is crucial for the precise engineering of thermoelectric materials.

## DISCUSSION

Within the classical PB model, the band effective mass serves as the sole parameter for modeling the band structure of semiconductors. This approximation is sufficiently adequate for describing electrical conductivity, as it relates to the properties of electrons within a narrow range near the Fermi level, exhibiting the local characteristics. However, thermoelectric transport properties, including the Seebeck coefficient and the Lorenz number, possess strong non-local characteristics, rendering this assumption inadequate for them. This feature indicates that the band structure deep within the valence or conduction band can significantly influence these parameters in certain semiconductor materials with low carrier concentrations; even their Fermi level remains within the bandgap. Here, we introduce a nonparabolicity factor *ξ* parameter derived from the limit of the Seebeck coefficient. Due to its direct correlation with thermoelectric parameters, the nonparabolicity factor *ξ* offers a more robust quantification of the effects of band nonparabolicity on thermoelectric transport. Notably, while the nonparabolicity factor *ξ* is derived within the isotropic Kane model, it can be effectively extended to more complex scenarios, such as anisotropic or degenerate valleys (as discussed in detail in Note S6). This adaptability allows *ξ* to serve as a robust metric for the nonparabolic description of energy bands in various materials. Furthermore, we can readily extend this approach to general band structures according to its definition, thereby introducing a new dimension to provide additional indicators into band shapes.

Accurate determination of lattice thermal conductivity is fundamental for understanding thermal transport mechanisms in thermoelectric materials. This is conventionally accomplished by subtracting the electronic thermal conductivity from the total thermal conductivity using the Wiedemann–Franz law (*κ*_L_ = *κ* – *LσT*), which strongly depends on accurately assessing *L*. For metallic materials, an empirical *L* value of 2.44 × 10^−8^ W Ω K^−2^ is commonly employed, which aligns with predictions from both the PB and Kane band models under heavy doping. However, this value significantly overestimates *L* for thermoelectric semiconductors, in which *L* may decrease to 1.49 × 10^−8^ W Ω K^−2^ in the parabolic model and 0.74 × 10^−8^ W Ω K^−2^ in the Kane model. Consequently, the electronic thermal conductivity may be overestimated by three times in extreme cases if an unreasonable Lorenz number is employed, ultimately resulting in a misguided lattice thermal conductivity. Furthermore, the temperature dependence of lattice thermal conductivity acts as a valuable indicator of phonon scattering mechanisms. For instance, a *T*^−1^ dependence typically indicates dominant acoustic phonon scattering, while a *T*^−1/2^ dependence suggests that point defect scattering is more significant. Thus, an inadequate estimate of *L* may mislead interpretations of thermal transport mechanisms. The *ξ*-modified solution for *L* offers a comprehensive framework for analysing lattice thermal conductivity and better capturing the complexities of phonon interactions in thermoelectric semiconductors, ultimately advancing our insights into their thermal transport.

In summary, this study underscores the essential role of incorporating band nonparabolicity into the analysis of thermoelectric properties. Our findings indicate that band nonparabolicity can result in considerable deviations from the predictions of the typical PB model, leading to overestimations of the maximum *PF* and inaccurate values for electronic thermal conductivity. By incorporating the nonparabolicity factor *ξ*, this study provides a quantitative framework for assessing the impact of band structure variations on thermoelectric performance. Notably, our findings demonstrate that nonparabolicity deep within the valence or conduction band significantly influences lightly doped semiconductors due to the non-local characteristics of the Seebeck coefficient and the Lorenz number, challenging conventional assumptions. Furthermore, a *ξ*-modified solution for *L* from experimental Seebeck coefficients is established, addressing the non-physical lattice thermal conductivity produced by the typical PB model. These insights indicate that a comprehensive understanding of band nonparabolicity should be integral to semiconductor design, guiding the development of advanced thermoelectric materials with tailored properties aimed at enhancing performance.

## MATERIALS AND METHODS

Full details of the first-principles calculations and experimental methods are provided in the online Supplementary file.

## Supplementary Material

nwaf216_Supplementary_File

## Notes

***Conflict of interest of statement.*** None declared.

## References

[1] Polman A, Knight M, Garnett EC et al. Photovoltaic materials: present efficiencies and future challenges. Science 2016; 352: aad4424. 10.1126/science.aad4424 27081076

[2] Shi J, Zhang J, Yang L et al. Wide bandgap oxide semiconductors: from materials physics to optoelectronic devices. Adv Mater 2021; 33: 2006230. 10.1002/adma.202006230 33797084

[3] Shi J, Starr MB, Wang X. Band structure engineering at heterojunction interfaces via the piezotronic effect. Adv Mater 2012; 24: 4683–91. 10.1002/adma.201104386 22549965

[4] Snyder GJ, Toberer ES. Complex thermoelectric materials. Nat Mater 2008; 7: 105–14. 10.1038/nmat2090 18219332

[5] Kim HS, Liu W, Ren Z. The bridge between the materials and devices of thermoelectric power generators. Energy Environ Sci 2017; 10: 69–85. 10.1039/C6EE02488B

[6] Imasato K, Kang SD, Snyder GJ. Exceptional thermoelectric performance in Mg_3_Sb_0.6_Bi_1.4_ for low-grade waste heat recovery. Energy Environ Sci 2019; 12: 965–71. 10.1039/C8EE03374A

[7] Park J, Dylla M, Xia Y et al. When band convergence is not beneficial for thermoelectrics. Nat Commun 2021; 12: 3425. 10.1038/s41467-021-23839-w 34103539 PMC8187731

[8] Shi X, Song S, Gao G et al. Global band convergence design for high-performance thermoelectric power generation in Zintls. Science 2024; 384: 757–62. 10.1126/science.adn7265 38753787

[9] Pei Y, Shi X, Lalonde A et al. Convergence of electronic bands for high performance bulk thermoelectrics. Nature 2011; 473: 66–9. 10.1038/nature09996 21544143

[10] Zhang Q, Liao B, Lan Y et al. High thermoelectric performance by resonant dopant indium in nanostructured SnTe. Proc Natl Acad Sci USA 2013; 110: 13261–6. 10.1073/pnas.1305735110 23901106 PMC3746939

[11] Heremans JP, Wiendlocha B, Chamoire AM. Resonant levels in bulk thermoelectric semiconductors. Energy Environ Sci 2012; 5: 5510–30. 10.1039/C1EE02612G

[12] Xiao Y, Wang D, Zhang Y et al. Band sharpening and band alignment enable high quality factor to enhance thermoelectric performance in n-type PbS. J Am Chem Soc 2020; 142: 4051–60. 10.1021/jacs.0c00306 32017566

[13] Lei J, Wuliji H, Ren Q et al. Exceptional thermoelectric performance in AB_2_Sb_2_ -type Zintl phases through band shaping. Energy Environ Sci 2024; 17: 1416–25. 10.1039/D3EE04164F

[14] Rowe DM . Thermoelectrics Handbook: Macro to Nano. New York: CRC Press, 2005, 243–62.

[15] Madsen GKH, Singh DJ. BoltzTraP: a code for calculating band-structure dependent quantities. Comput Phys Commun 2006; 175: 67–71. 10.1016/j.cpc.2006.03.007

[16] Madsen GKH, Carrete J, Verstraete MJ. BoltzTraP2, a program for interpolating band structures and calculating semi-classical transport coefficients. Comput Phys Commun 2018; 231: 140–5. 10.1016/j.cpc.2018.05.010

[17] Zhu J, Zhang X, Guo M et al. Restructured single parabolic band model for quick analysis in thermoelectricity. npj Comput Mater 2021; 7: 116. 10.1038/s41524-021-00587-5

[18] Pei Y, Lalonde AD, Wang H et al. Low Effective mass leading to high thermoelectric performance. Energy Environ Sci 2012; 5: 7963. 10.1039/c2ee21536e

[19] Wang H, Pei Y, Lalonde AD et al. Heavily doped p-type PbSe with high thermoelectric performance: an alternative for PbTe. Adv Mater 2011; 23: 1366–70. 10.1002/adma.201004200 21400597

[20] Zhou M, Gibbs ZM, Wang H et al. Optimization of thermoelectric efficiency in SnTe: the case for the light band. Phys Chem Chem Phys 2014; 16: 20741–8. 10.1039/C4CP02091J 25162449

[21] Li A, Hu C, He B et al. Demonstration of valley anisotropy utilized to enhance the thermoelectric power factor. Nat Commun 2021; 12: 5408. 10.1038/s41467-021-25722-0 34535648 PMC8448840

[22] Kane EO . Band structure of indium antimonide. J Phys Chem Solids 1957; 1: 249–61. 10.1016/0022-3697(57)90013-6

[23] Naithani H, Dasgupta T. Critical analysis of single band modeling of thermoelectric materials. ACS Appl Energy Mater 2020; 3: 2200–13. 10.1021/acsaem.9b02015

[24] Mecholsky NA, Resca L, Pegg IL et al. Theory of band warping and its effects on thermoelectronic transport properties. Phys Rev B 2014; 89: 155131. 10.1103/PhysRevB.89.155131

[25] Usui H, Suzuki K, Kuroki K et al. Large Seebeck effect in electron-doped FeAs_2_ driven by a quasi-one-dimensional pudding-mold-type band. Phys Rev B 2013; 88: 75140. 10.1103/PhysRevB.88.075140

[26] Li X, Sheng Y, Wu L et al. Defect-mediated Rashba engineering for optimizing electrical transport in thermoelectric BiTeI. npj Comput Mater 2020; 6: 107. 10.1038/s41524-020-00378-4

[27] Chen X, Parker D, Singh DJ. Importance of non-parabolic band effects in the thermoelectric properties of semiconductors. Sci Rep 2013; 3: 3168. 10.1038/srep03168 24196778 PMC3819605

[28] Xing G, Sun J, Li Y et al. Electronic fitness function for screening semiconductors as thermoelectric materials. Phys Rev Mater 2017; 1: 65405. 10.1103/PhysRevMaterials.1.065405

[29] Shi H, Parker D, Du M et al. Connecting thermoelectric performance and topological-insulator behavior: Bi_2_Te_3_ and Bi_2_Te_2_Se from first principles. Phys Rev Appl 2015; 3: 14004. 10.1103/PhysRevApplied.3.014004

[30] Gibbs ZM, Ricci F, Li G et al. Effective mass and Fermi surface complexity factor from ab initio band structure calculations. npj Comput Mater 2017; 3: 8. 10.1038/s41524-017-0013-3

[31] Hsieh TH, Lin H, Liu J et al. Topological crystalline insulators in the SnTe material class. Nat Commun 2012; 3: 982. 10.1038/ncomms1969 22864575

[32] Rogers LM . Valence band structure of SnTe. J Phys D: Appl Phys 1968; 1: 845–52. 10.1088/0022-3727/1/7/304

[33] Strauss AJ, Brebrick RF. Anomalous thermoelectric power as evidence for two-valence bands in SnTe. Phys Rev 1963; 131: 104–10. 10.1103/PhysRev.131.104

[34] Zhang X, Bu Z, Shi X et al. Electronic quality factor for thermoelectrics. Sci Adv 2020; 6: eabc0726. 10.1126/sciadv.abc0726 33188018 PMC7673762

[35] Jiang Q, Li G, Wang X et al. Enhanced thermoelectric properties for eco-friendly CaTiO_3_ by band sharpening and atomic-scale defect phonon scattering. Mater Today Energy 2024; 44: 101655. 10.1016/j.mtener.2024.101655

[36] Putatunda A, Singh DJ. Lorenz number in relation to estimates based on the Seebeck coefficient. Mater Today Phys 2019; 8: 49–55. 10.1016/j.mtphys.2019.01.001

[37] Kim H, Gibbs ZM, Tang Y et al. Characterization of Lorenz number with Seebeck coefficient measurement. APL Mater 2015; 3: 41506. 10.1063/1.4908244

[38] Cahill DG, Watson SK, Pohl RO. Lower limit to the thermal conductivity of disordered crystals. Phys Rev B 1992; 46: 6131–40. 10.1103/PhysRevB.46.6131 10002297

[39] Agne MT, Hanus R, Snyder GJ et al. Minimum thermal conductivity in the context of diffuson-mediated thermal transport. Energy Environ Sci 2018; 11: 609–16. 10.1039/C7EE03256K

[40] Qin H, Liu Y, Zhang Z et al. Improved thermoelectric performance of p-type Bi_0.5_Sb_1.5_Te_3_ through Mn doping at elevated temperature. Mater Today Phys 2018; 6: 31–7. 10.1016/j.mtphys.2018.07.002

[41] Zhang Q, Guo Z, Wang R et al. High-performance thermoelectric material and module driven by medium-entropy engineering in SnTe. Adv Funct Mater 2022; 32: 2205458. 10.1002/adfm.202205458

[42] You L, Liu Y, Li X et al. Boosting the thermoelectric performance of PbSe through dynamic doping and hierarchical phonon scattering. Energy Environ Sci 2018; 11: 1848–58. 10.1039/C8EE00418H

[43] Thompson DR, Liu C, Yang J et al. Rare-earth free p-type filled skutterudites: mechanisms for low thermal conductivity and effects of Fe/Co ratio on the band structure and charge transport. Acta Mater 2015; 92: 152–62. 10.1016/j.actamat.2015.03.032

