# Supplementary material for: Revisiting thermoelectric transport properties through a band nonparabolicity factor
Source: Natl Sci Rev. 2025 May 31;12(8):nwaf216. doi: 10.1093/nsr/nwaf216 (PMC12236334; doi:10.1093/nsr/nwaf216)
Supplement: nwaf216_Supplementary_File [file nwaf216_supplementary_file.pdf]

Supplementary Information

Revisiting Thermoelectric Transport Properties Through a Band Nonparabolicity Factor

Jianbo Zhu[a], Ming Liu[a], Xingyan Dong[a], Jingyu Li[b,c], Peng-Fei Liu[b,c], Xin Chen[d], Zihang Liu[a], Yongsheng Zhang[d,*], Fengkai Guo[a,*] and Jiehe Sui[a,*]

[a]State Key Laboratory of Precision Welding & Joining of Materials and Structures, Harbin Institute of Technology, Harbin 150001, China;
[b]Institute of High Energy Physics, Chinese Academy of Sciences, Beijing 100049, China;
[c]Spallation Neutron Source Science Center, Dongguan 523803, China;
[d]Advanced Research Institute of Multidisciplinary Sciences, Qufu Normal University, Qufu 273165, China

***Corresponding authors**. E-mails: yshzhang@qfnu.edu.cn; fkguo@hit.edu.cn; suijiehe@hit.edu.cn

1. Materials and Methods

First principles calculation

The electronic structure calculations were performed using the projector augmented wave (PAW) method [1] implemented in the Vienna Ab Initio Simulation Package (VASP) [2]. Structural optimizations and lattice relaxations were conducted until the forces acting on all atoms were less than 0.001 eV $Å^{-1}$. A plane-wave cutoff energy of 550 eV was employed for self-consistent field calculations to ensure convergence. For most semiconductor materials, including Mg_3Sb_2, $CaMg_2Bi_2$, PbSe, $CoSb_3$, ZrNiSn, and NbFeSb, the generalized gradient approximation (GGA) with the Perdew-Burke-Ernzerhof (PBE) functional [3] was utilized. For materials containing heavy elements, such as SnTe, PbTe, Bi_2Te_3, and Sb_2Te_3, spin-orbit coupling (SOC) effects were explicitly included to account for relativistic interactions. The local density approximation (LDA) functional [4] has been employed for Bi_2Te_3 and Sb_2Te_3, as previously reported, differing from other materials. This choice is supported by extensive prior research which demonstrates that LDA provides better agreement with experimental results for these specific compounds [5]. For Si, Ge, GaAs, Mg_2Si, and Mg_2Sn, the hybrid functional HSE06 [6] was employed to achieve better accuracy in the electronic band structure calculations. A dense *k*-mesh (details provided in **Supplementary Table S1**) combined with the tetrahedron method was used to obtain high-quality density-of-states, ensuring accurate extraction of effective masses.

The electronic transport properties of SnTe were computed using the BoltzTrap2 package [7]. The electronic relaxation time was further calculated using the AMSET package [8], considering acoustic phonon scattering as the dominant scattering mechanism. To minimize numerical instabilities arising from band crossings during derivative evaluations, a dense 80×80×80 *k*-mesh was employed. Additionally, since PBE underestimates the bandgap of semiconductors, the bandgap of SnTe was scissored to match the experimental value of 0.18 eV [9]. The power factor dispersion is defined as $PF_s(E, k; \mathrm{T}) = \Sigma_i\, \sigma_s(\varepsilon_i, k; \mathrm{T})\cdot[S(E; \mathrm{T})]^2\cdot w_F(\varepsilon_i - E; \mathrm{T})$, where ε_i represents the *i*-th eigenvalue, and k is the wavevector. The term σ_s denotes the electronic transport distribution function, and w_F is the derivative of the equilibrium Fermi-Dirac distribution. A key feature of $PF_s(E, k; \mathrm{T})$ is that integration over the k-space at a specific energy E results in the corresponding $PF(E; T)$, providing a comprehensive representation of the power factor distribution in reciprocal space. Here, $\sigma_s(\varepsilon_i, k; \mathrm{T})$ was computed using the submodule of the BoltzTrap2 package,

incorporating electronic relaxation times from AMSET. Then, PF_s for each (E, k) sampling point were derived by summing all eigenvalue contributions using the interpolated $S(E; T)$ and weights w_F.

Sample synthesis and characterization

High-purity Sn shot (Alfa Aesar, 99.999%), Te shot (Alfa Aesar, 99.999%), and Be pieces (Alfa Aesar, 99.99%) were weighed according to the stoichiometry of $Sn_{1.03}Te$ and $Sn_{1.02}Be_{0.01}Te$. Strict protective measures were implemented during processing due to the hazardous nature of Be, which can cause severe health issues such as lung disease and cancer. The material was handled carefully to prevent human contact, and the protective atmosphere was rigorously managed. The materials were loaded into quartz tubes, which were thoroughly cleaned and then sealed under high vacuum (< 10^{-2} Pa). The sealed tubes were gradually heated to 723 K over 10 hours, followed by heating to 1423 K over 6 hours, where they were maintained for 8 hours before being quenched in cold water. The quenched ingots were annealed at 923 K for 72 hours to ensure homogeneity, then ground into fine powders in an agate mortar for XRD analysis and hot pressing. Pellets were prepared by hot pressing the powders at 923 K for 90 minutes under uniaxial pressure of 100 MPa in a high vacuum environment (< 5×10^{-3} Pa). The resulting dense samples (> 98% of theoretical density) were approximately 12.7 mm in diameter and 9.0 mm in height.

Phase composition was determined using powder XRD with Cu K_α radiation (wavelength λ = 1.5406 Å), as shown in **Supplementary Figure S6**. Disk-shaped samples (12.7 mm diameter, 1.5 mm thickness) were used to measure thermal diffusivity (D) on a laser flash apparatus (Netzsch LFA 427). Heat capacity (C_p) values were adopted from prior literature, and the thermal conductivity (κ) was calculated as $\kappa = D \times C_p \times \rho$, where ρ is the sample density determined using the Archimedes method. Samples with dimensions of $2 \times 2 \times 12$ mm^3 were prepared for simultaneous measurements of the Seebeck coefficient (S) and electrical conductivity (σ) using ZEM-3 (Ulvac-Riko, Inc. Japan). Thermoelectric properties of samples are shown in **Supplementary Figure S7**. The Debye-Cahill limit [10]of the lattice thermal conductivity is calculated using the formula $\kappa_{min} = \left(\frac{\pi}{6}\right)^{1/3} k_B n^{2/3} \sum_i v_i \left(\frac{T}{\Theta_i}\right)^2 \int_0^{\Theta_i/T} \frac{x^3 e^x}{(e^x-1)^2} dx$, where the sum is taken over the three sound modes (two transverse and one longitudinal) with sound velocities v_i and Debye temperatures Θ_i, and n is the number density of atoms.

2. Supplementary Figures

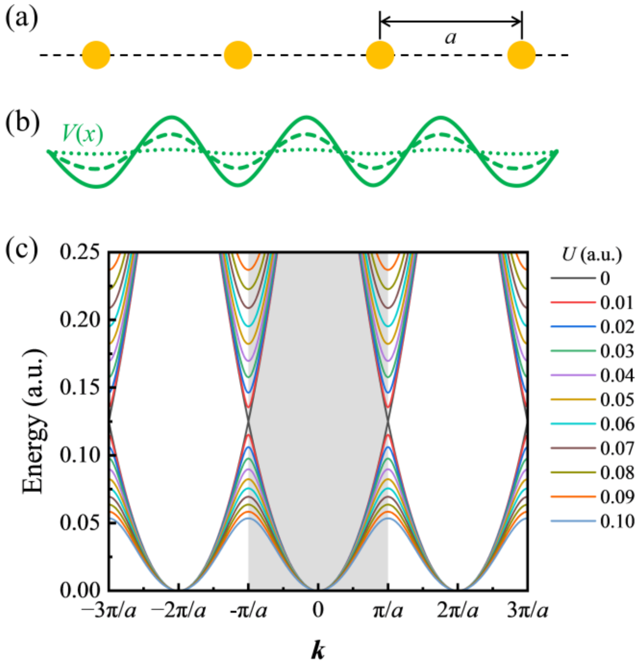

Figure S1. One-dimensional atomic chain model. **(a)** Schematic diagram of a one-dimensional atomic chain with an interatomic distance of a. **(b)** Schematic representation of a cosine periodic potential $V(\mathrm{x}) = 2U\cos(2\pi/a \cdot x)$, where the parameter U reflects the strength of interatomic interactions. **(c)** The band structure in one-dimensional reciprocal space.

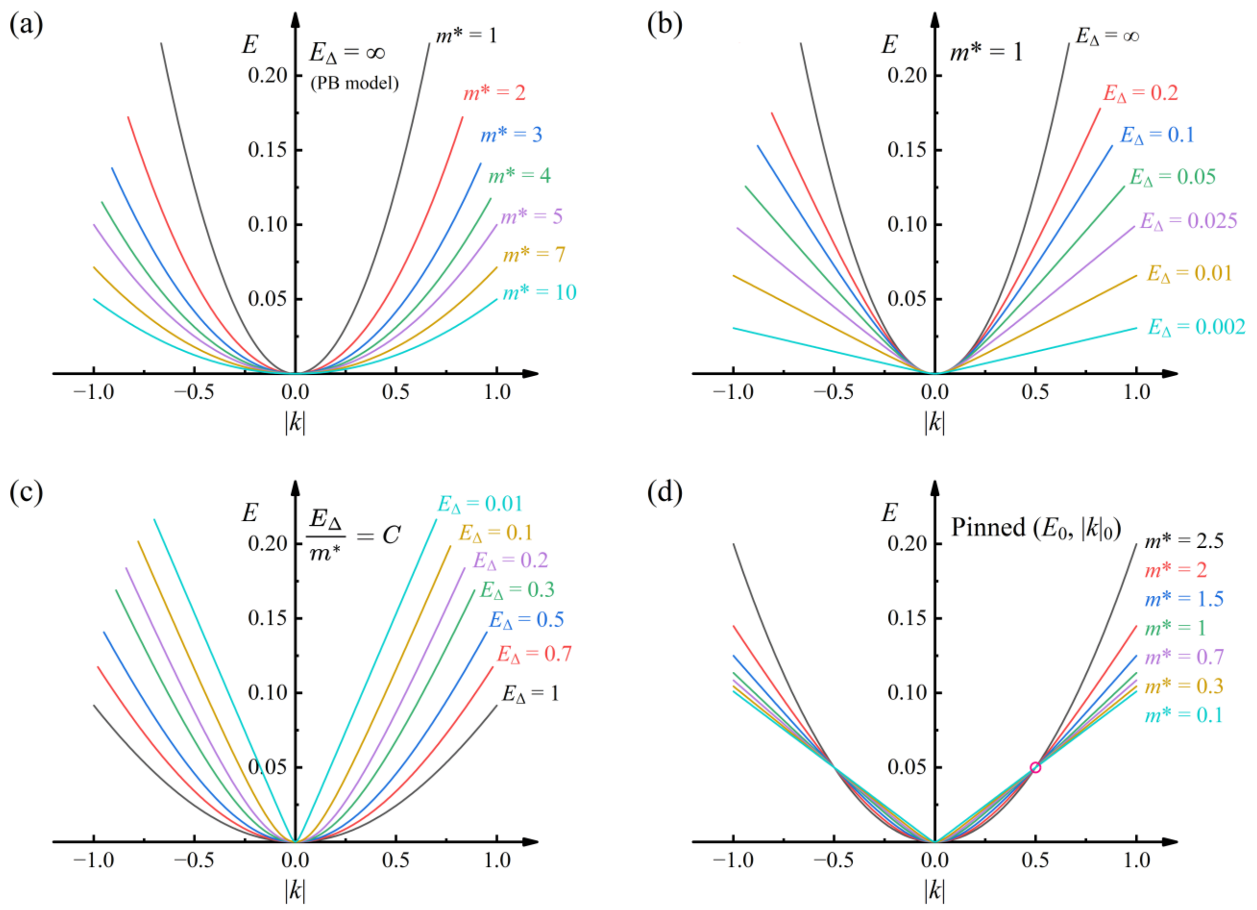

Figure S2. Kane model dispersion with varying parameters (m^*, E_Δ). **(a)** $E_\Delta = \infty$, corresponding to a parabolic band where nonparabolic effects are absent. **(b)** E_Δ varies while m^* is fixed at 1. **(c)** The ratio E_Δ/m^* is fixed as a constant ($C = 0.2$). **(d)** The band is constrained to pass through a fixed point (E_0, $|k|_0$), highlighting the flexibility of the Kane model. All quantities are in ***atomic units*** (E_h for energy, $\hbar/a_0$ for momentum, m_e for mass).

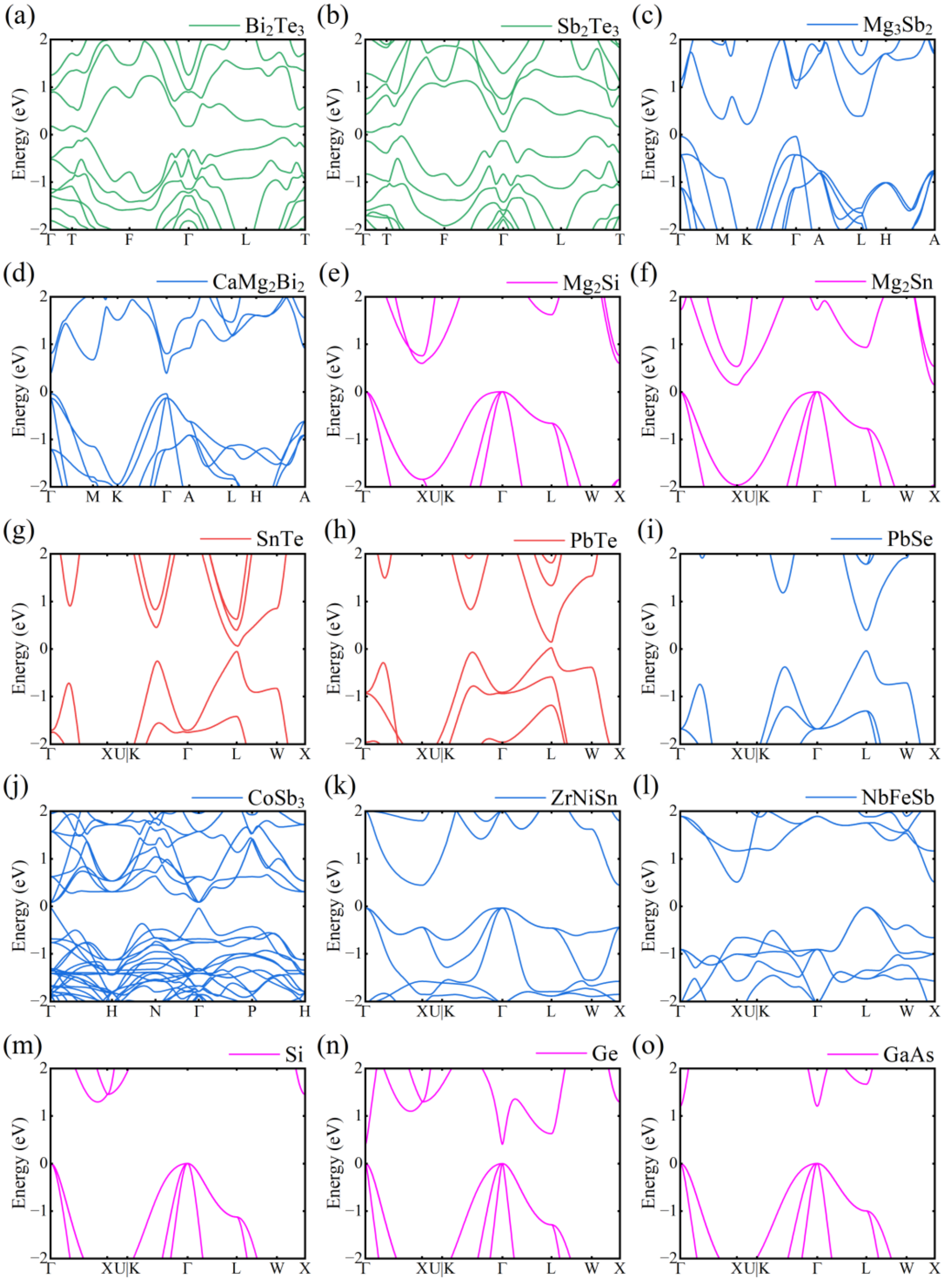

Figure S3. Band structures of some typical functional semiconductor materials. The solid lines with varying colors represent the distinct exchange-correlation functionals applied (see **Table S1** for details). **(a)** Bi_2Te_3, **(b)** Sb_2Te_3, **(c)** Mg_3Sb_2, **(d)** $CaMg_2Bi_2$, **(e)** Mg_2Si, **(f)** Mg_2Sn, **(g)** SnTe, **(h)** PbTe, **(i)** PbSe, **(j)** $CoSb_3$, **(k)** ZrNiSn, **(l)** NbFeSb, **(m)** Si, **(n)** Ge, **(o)** GaAs.

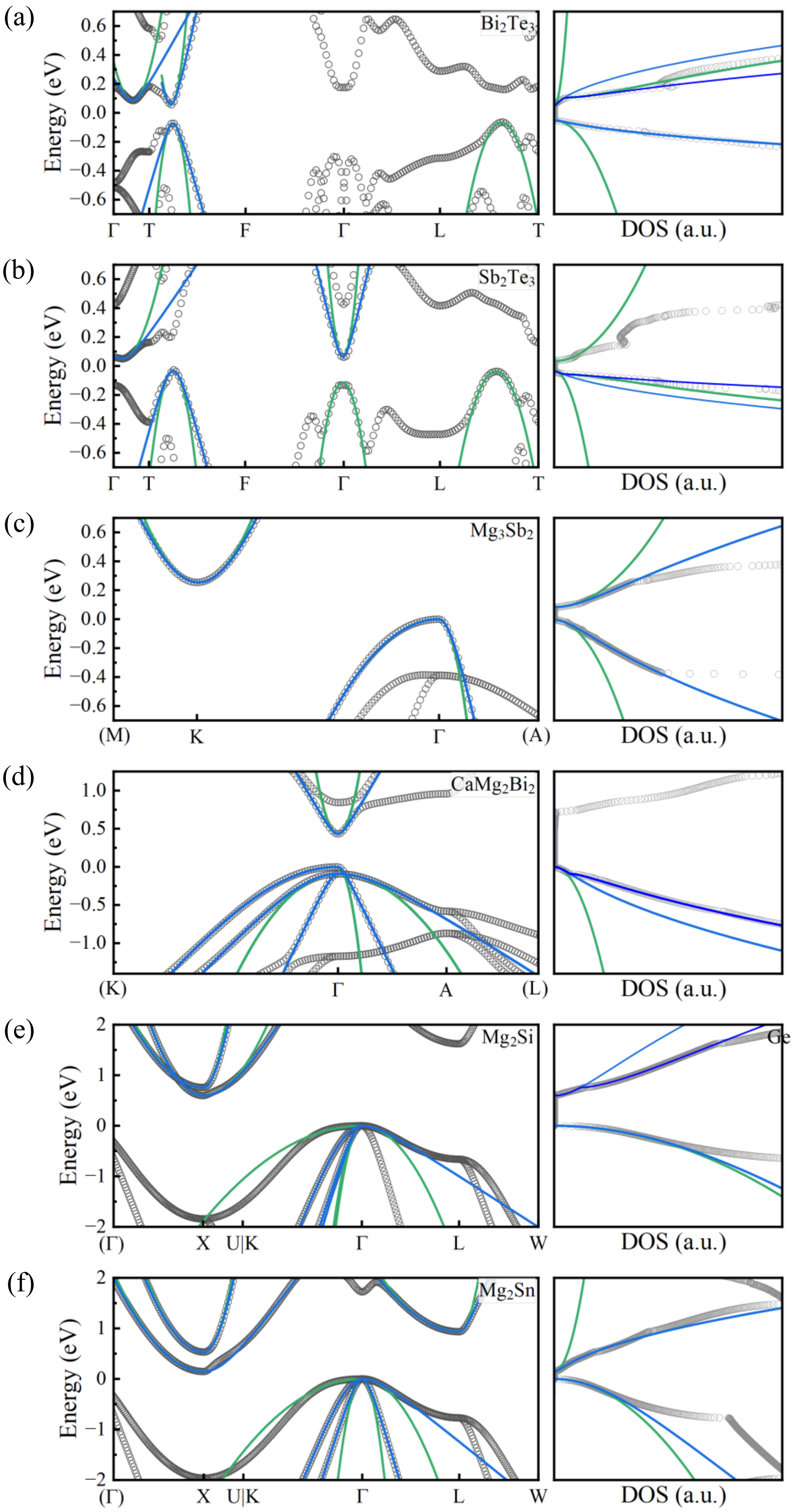

(a)
Bi2Te3
Energy (eV)
0.6
0.4
0.2
0.0
−0.2
−0.4
−0.6
Γ T F Γ L T
DOS (a.u.)
(b)
Sb2Te3
Energy (eV)
Γ T F Γ L T
DOS (a.u.)
(c)
Mg3Sb2
Energy (eV)
(M) K Γ (A)
DOS (a.u.)
(d)
CaMg2Bi2
Energy (eV)
1.0
0.5
0.0
−0.5
−1.0
(K) Γ A (L)
DOS (a.u.)
(e)
Mg2Si
Ge
Energy (eV)
2
1
0
−1
−2
(Γ) X U|K Γ L W
DOS (a.u.)
(f)
Mg2Sn
Energy (eV)
(Γ) X U|K Γ L W
DOS (a.u.)

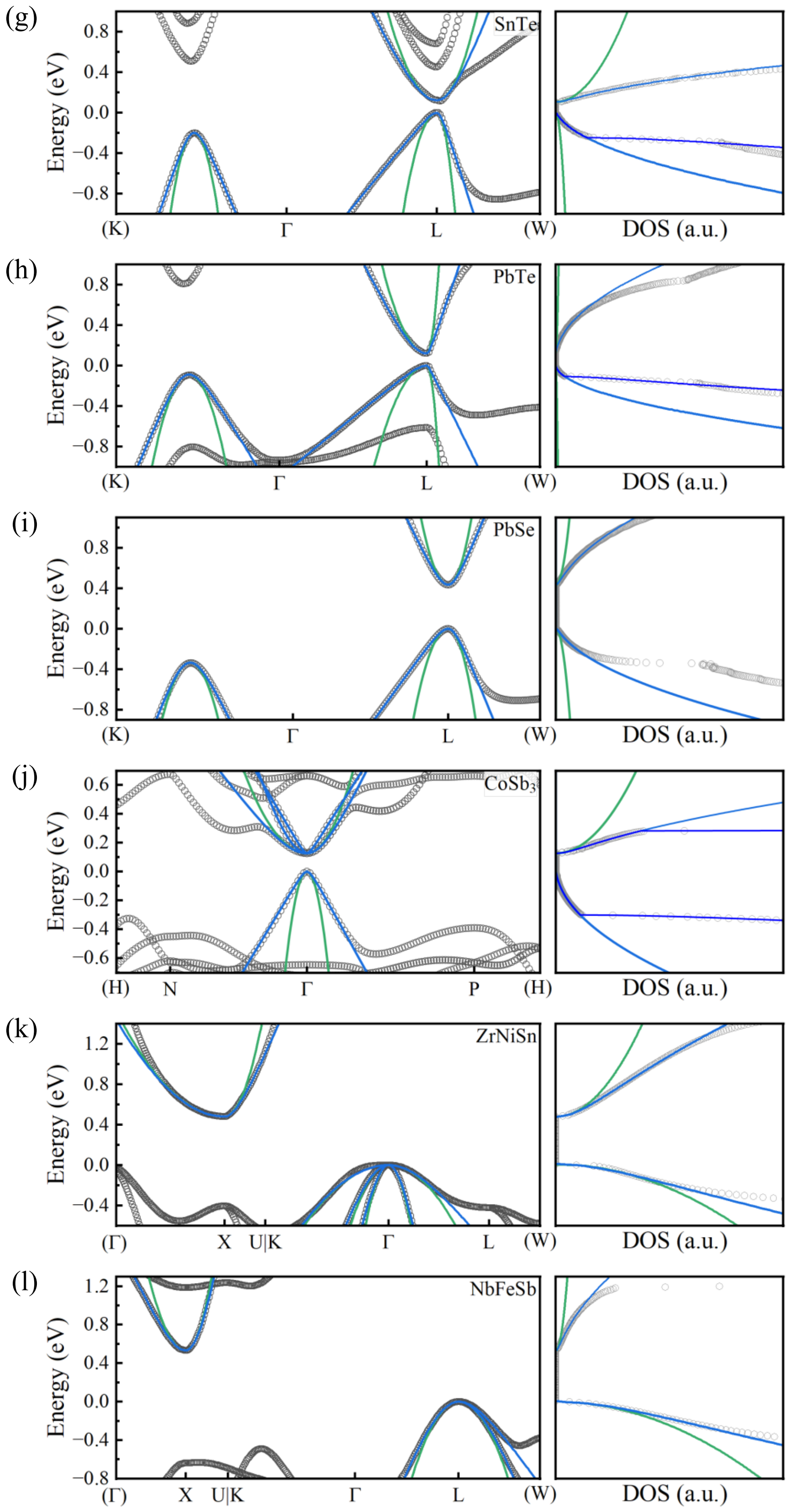
(g)
SnTe
Energy (eV)
0.8
0.4
0.0
−0.4
−0.8
(K)
Γ
L
(W)
DOS (a.u.)
(h)
PbTe
Energy (eV)
(K)
Γ
L
(W)
DOS (a.u.)
(i)
PbSe
Energy (eV)
(K)
Γ
L
(W)
DOS (a.u.)
(j)
CoSb3
Energy (eV)
0.6
0.4
0.2
0.0
−0.2
−0.4
−0.6
(H)
N
Γ
P
(H)
DOS (a.u.)
(k)
ZrNiSn
Energy (eV)
1.2
0.8
0.4
0.0
−0.4
(Γ)
X
U|K
Γ
L
(W)
DOS (a.u.)
(l)
NbFeSb
Energy (eV)
1.2
0.8
0.4
0.0
−0.4
−0.8
(Γ)
X
U|K
Γ
L
(W)
DOS (a.u.)

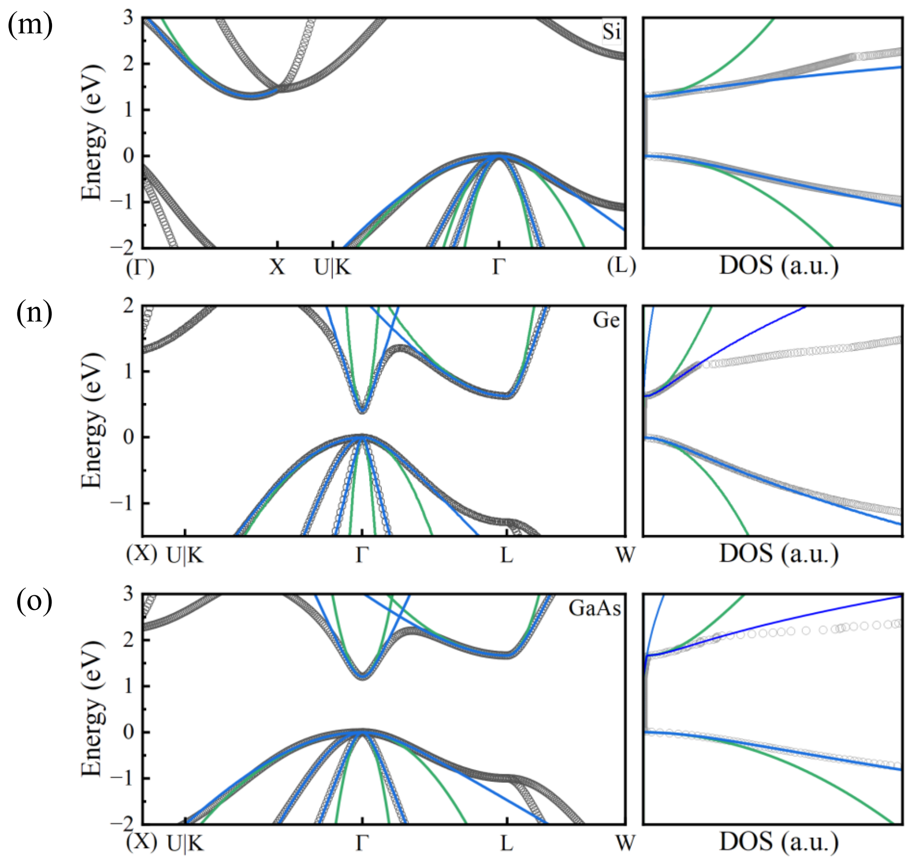

Figure S4. Fitting of band extremes and density of states (DOS) using the parabolic (green) and Kane (blue) band model. **(a)** Bi_2Te_3, **(b)** Sb_2Te_3, **(c)** Mg_3Sb_2, **(d)** $CaMg_2Bi_2$, **(e)** Mg_2Si, **(f)** Mg_2Sn, **(g)** SnTe, **(h)** PbTe, **(i)** PbSe, **(j)** $CoSb_3$, **(k)** ZrNiSn, **(l)** NbFeSb, **(m)** Si, **(n)** Ge, **(o)** GaAs.

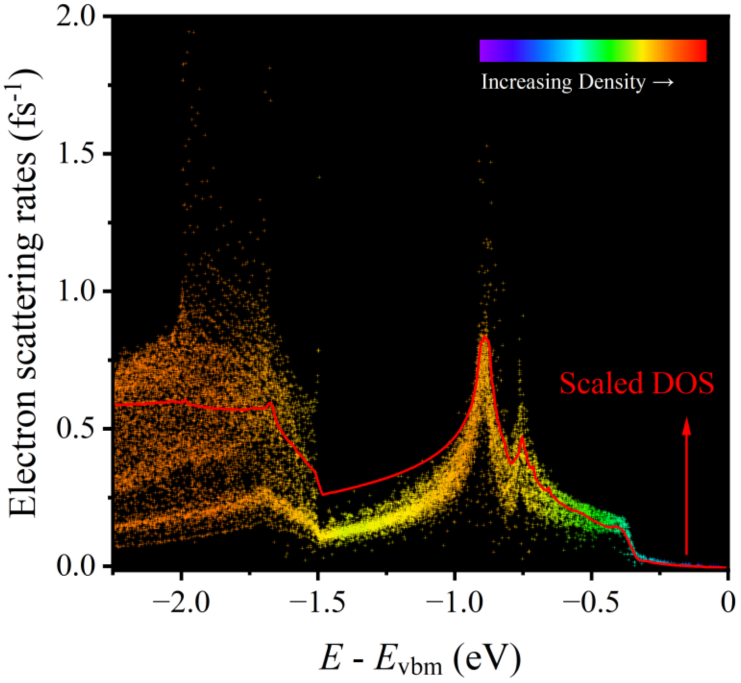

Figure S5. Electrical scattering rates and scaled density-of-states (DOS) of SnTe.

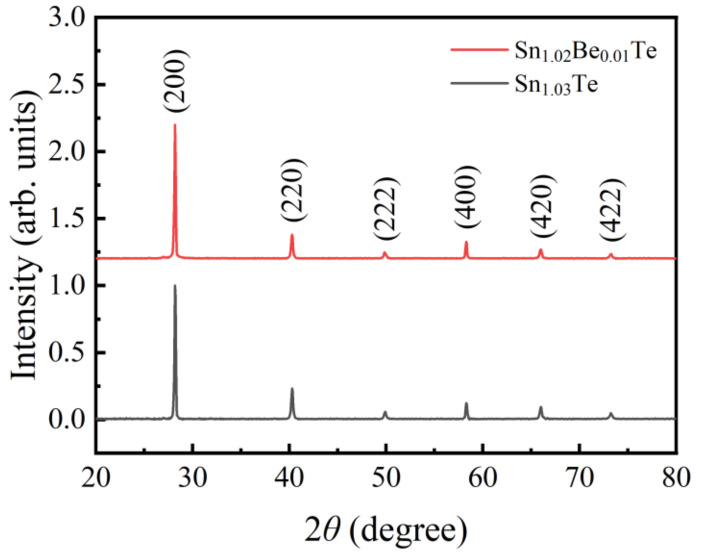

Figure S6. Powder XRD patterns of undoped and Be-doped SnTe.

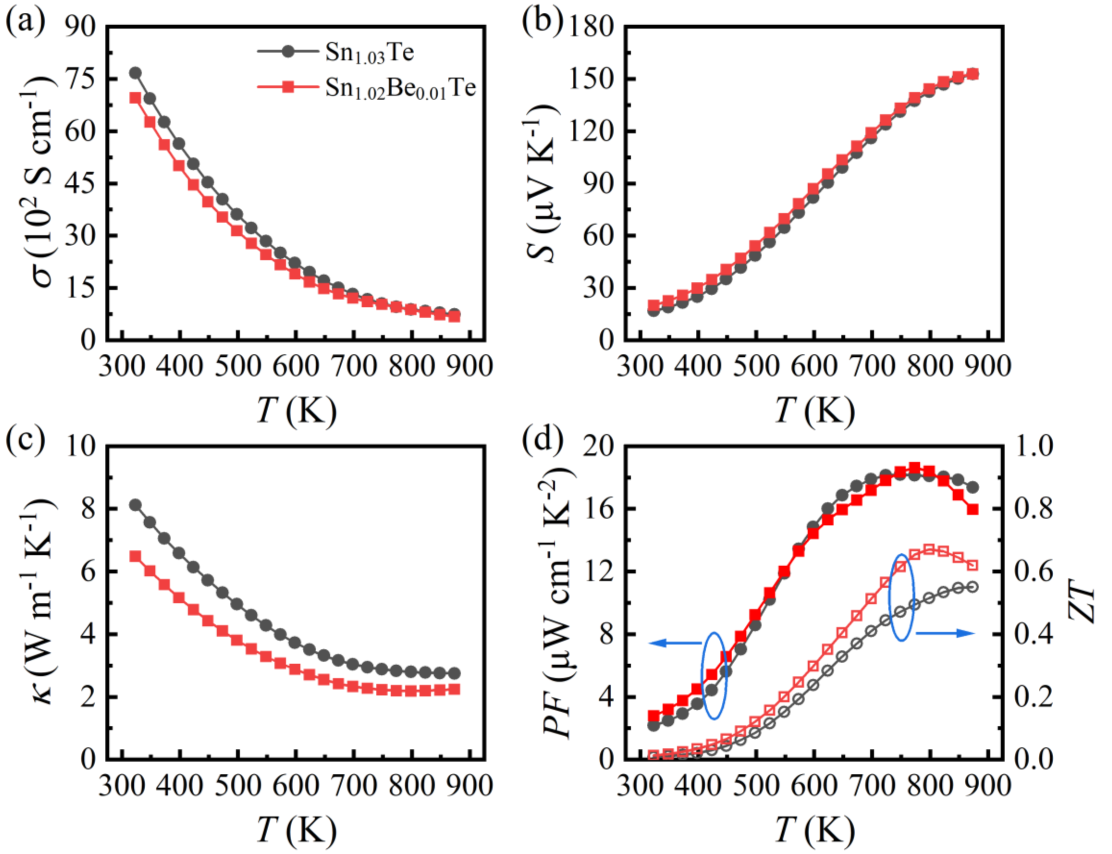

Figure S7. Thermoelectric properties of Be-doped SnTe. **(a)** Conductivity (*σ*); **(b)** Seebeck coefficient (*S*); **(c)** Thermal conductivity (*κ*); **(d)** Power factor (*PF*) and figure-of-merit (*ZT*).

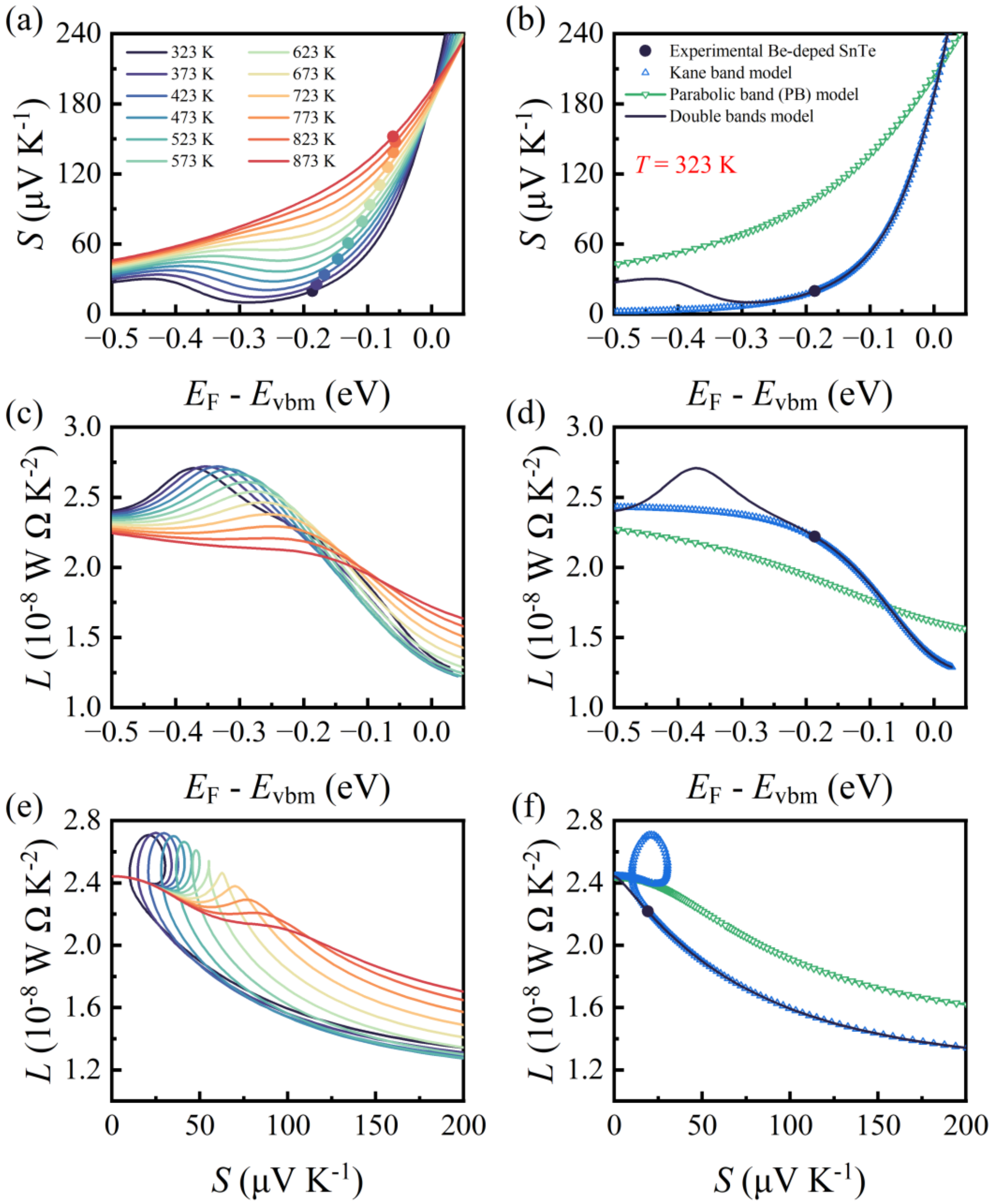

Figure S8. Seebeck coefficients and Lorenz numbers of SnTe under the double valence bands model. Dependence of **(a)** Seebeck coefficients and **(c)** Lorenz numbers on Fermi level at 323 ~ 873 K. Model comparison of **(b)** Seebeck coefficients and **(d)** Lorenz numbers at 323 K. **(e)** Relationship between Lorenz numbers and Seebeck coefficients at 323 ~ 873 K. **(f)** Model comparison at 323 K. The solid markers represent experimental data for the Be-doped SnTe sample, where the Fermi levels are determined from the Seebeck coefficients at different temperatures.

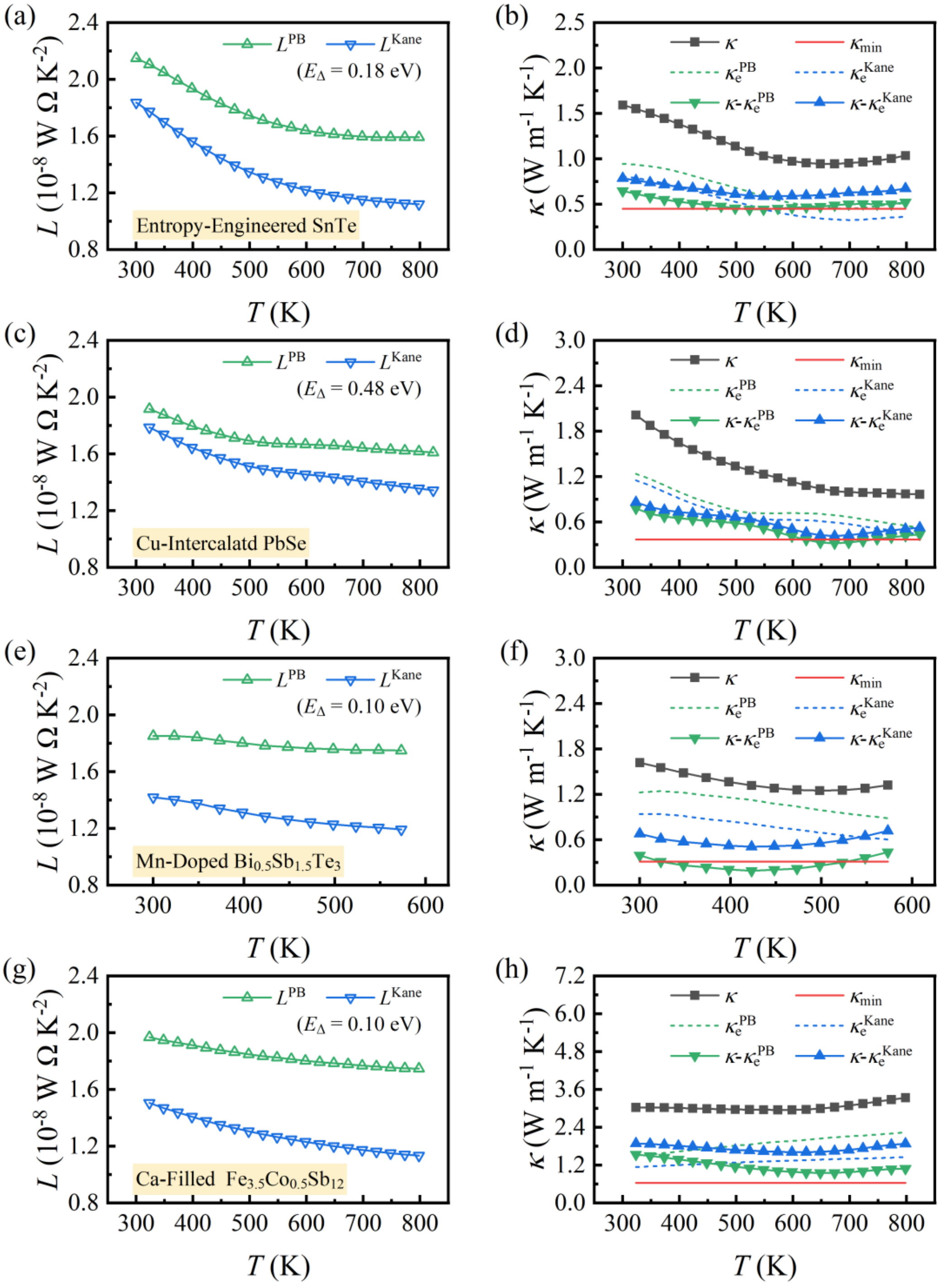

Figure S9. Comparison of Lorenz number (L), electronic and lattice thermal conductivity (κ_e and κ-κ_e) under the parabolic (PB) and Kane band model. **(a)** and **(b)** are Lorenz number and thermal conductivities for entropy-engineered SnTe, respectively; **(c)** and **(d)** for Cu-intercalated PbSe; **(e)** and **(f)** for Mn-doped $Bi_{0.5}Sb_{1.5}Te_3$; **(g)** and **(h)** for Sample Ca-filled $Fe_{3.5}Co_{0.5}Sb_{12}$.

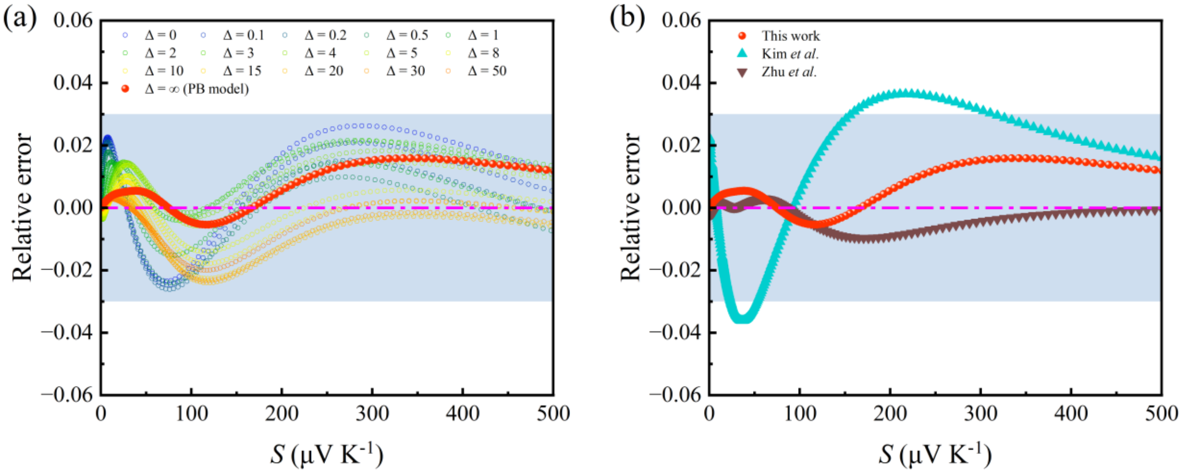

Figure S10. Comparison of relative errors for the L-S analytical model. **(a)** Relative errors of the proposed analytical model for different values of Δ, demonstrating deviations below 3% as stated in the main text. **(b)** Comparison between the proposed model, the empirical analytical model for the Lorenz number by H. Kim et al. (APL Mater., 3, 041506, 2015), and the previously proposed reconstructed parabolic band model (Zhu et al., Npj Comput. Mater., 7, 116, 2021) under the ideal parabolic band model.

3. Supplementary Tables

Table S1. Details for DFT calculations and fitting parameters.

Materials	K-mesh	Exchange-Correlation Functionals	Density-of-state Effective Mass in m_e (E_Δ in eV)			
			CB1	VB1	CB2	VB2
Bi_2Te_3	40×40×40	LDA+SOC	0.134 (0.0976)	0.383 (0.0976)	0.673 (0.4513)	—
Sb_2Te_3	40×40×40	LDA+SOC	—	0.256 (0.1090)	—	0.675 (0.1291)
Mg_3Sb_2	45×45×45	PBE	0.663 (1.3500)	0.439 (0.8127)	—	—
$CaMg_2Bi_2$	45×45×45	PBE	—	0.465 (0.8922)	—	0.545 (1.7592)
Mg_2Si	80×80×80	HSE06	0.897 (4.6982)	1.938 (30)	0.929 (22.3654)	—
Mg_2Sn	80×80×80	HSE06	0.505 (0.5941)	1.379 (17.5286)	—	—
SnTe	96×96×96	PBE+SOC	0.517 (0.3024)	0.130 (0.1594)	—	1.799 (1.5231)
PbTe	96×96×96	PBE+SOC	0.094 (0.1386)	0.074 (0.0395)	—	1.840 (0.4010)
PbSe	80×80×80	PBE	0.321 (0.4832)	0.297 (0.2930)	—	—
$CoSb_3$	52×52×52	PBE	0.362 (0.4249)	0.035 (0.0991)	7.730 (25)	1.476 (0.5540)
ZrNiSn	80×80×80	PBE	1.588 (2.6027)	2.991 (10)	—	—
NbFeSb	80×80×80	PBE	0.622 (0.7014)	4.141 (2.5352)	—	—
Si	80×80×80	HSE06	1.073 (0.8709)	1.305 (3.5282)	—	—
Ge	80×80×80	HSE06	0.025 (0.4034)	0.875 (2.4173)	0.607 (2.4599)	—
GaAs	80×80×80	HSE06	0.083 (1.2058)	1.418 (3.5579)	0.761 (1.9253)	—

4. Supplementary Notes

Note S1. One-Dimensional Atomic Chain Model in a Periodic Potential

In a crystalline material, the electronic potential energy $V(x)$ is intrinsically periodic, mirroring the periodicity of the lattice structure. For a one-dimensional atomic chain with a lattice constant a, the periodicity condition can be expressed as $V(x + a) = V(x)$. A key method for analyzing periodic functions is the Fourier series, allowing us to express $V(x)$ as **Eq. S1**,

$$V(x) = \sum_G V_G \exp(iGx) \tag{S1}$$

where V_G denotes the expansion coefficient corresponding to the reciprocal lattice vector G. As the potential energy is a real function, it can be stated that $V_G = V_{-G}$. By incorporating Eq. S1 into the Schrödinger equation and expanding the wave function in terms of a basis of plane wave functions, we can obtain **Eq. S2**, known as the central equation,

$$(\lambda_k - E)C_k + \sum_G V_G C_{k-G} = 0,\ \ \lambda_k = \frac{\hbar^2 k^2}{2m_e} \tag{S2}$$

where $\lambda_k = \hbar^2 k^2/2m_e$, k is the wave vector, E is the energy, and m_e is the mass of the electron, C_k and C_{k-G} are expansion coefficients of the wave function. For the purpose of simplification, we focus on the first-order approximation, where $V_G = U$ for $G = \pm g$ and $V_G = 0$ otherwise, with g defined as the shortest G (i.e. $2\pi/a$). Consequently, $V(x)$ can be expressed in a simplified form as **Eq. S3**,

$$V(x) = U\exp(igx) + U\exp(-igx) = 2U\cos(gx) \tag{S3}$$

Considering a finite cutoff wave vector (with $10\cdot g$ adopted in this work), **Eq. S2** becomes a finite-dimensional eigenvalue problem of matrix A as **Eq. S4**,

$$A = \begin{bmatrix} \ddots & \vdots & \vdots & \vdots & \vdots & \vdots & ⋰ \\ \cdots & \lambda_{k-2g} & U & 0 & 0 & 0 & \cdots \\ \cdots & U & \lambda_{k-g} & U & 0 & 0 & \cdots \\ \cdots & 0 & U & \lambda_k & U & 0 & \cdots \\ \cdots & 0 & 0 & U & \lambda_{k+g} & U & \cdots \\ \cdots & 0 & 0 & 0 & U & \lambda_{k+2g} & \cdots \\ ⋰ & \vdots & \vdots & \vdots & \vdots & \vdots & \ddots \end{bmatrix} \tag{S4}$$

By varying the wave vector k across the interval from -1/2·g to 1/2·g and solving for the eigenvalues, we can obtain the band structure within the first Brillouin zone under the periodic potential, as shown in **Figure S1**.

Note S2. Parameter E_Δ vs. Bandgap E_g

The dispersion relation of the Kane model, originally derived from *k·p* theory, is expressed as:

$$E\left(1+\frac{E}{E_\Delta}\right)=\frac{1}{2}\frac{\hbar^2|k|^2}{m^*} \quad \text{(S5)}$$

This analytic model has two distinct features:

- The dispersion comprises two symmetric hyperbolic branches about E = -1/2 E_Δ, corresponding to the conduction band and valence band.
- The energy difference between the minimum of the upper branch and the maximum of the lower branch is exactly equal to E_Δ, which also represents the energy gap (E_g) between the conduction band and the valence band.

In this work, we bypass the complex physical properties of the Kane model and isolate the analytic form of a single branch to fit real band structures. In other words, we suggest using **Eq. S6** (obtained by solving E from **Eq. S5**) as an alternative to the simple parabolic band model, where E_Δ and m^* are fitting parameters:

$$E=-\frac{E_\Delta}{2}+\sqrt{\left(\frac{E_\Delta}{2}\right)^2+\frac{E_\Delta}{2}\frac{\hbar^2|k|^2}{m^*}}=\frac{1}{2}\frac{\hbar^2|k|^2}{m^*}\cdot\frac{1}{\frac{1}{2}+\sqrt{\frac{1}{4}+\frac{\hbar^2|k|^2}{2m^*E_\Delta}}} \quad \text{(S6)}$$

In practice, the anisotropy of the band structure and orbital degeneracy can complicate the parameter fitting process. Alternatively, the density of states $g(E)$ can also be used to fit E_Δ and m^* via the following relation:

$$g(E)=\frac{(2m_d^*)^{3/2}}{2\pi^2\hbar^3}E^{1/2}\cdot\left(1+\frac{E}{E_\Delta}\right)^{1/2}\left(1+2\frac{E}{E_\Delta}\right) \quad \text{(S7)}$$

Both **Eq. S6** and **Eq. S7** fully encompass the parabolic band model as a limiting case when E_Δ approaches infinity. The introduction of the additional parameter E_Δ is expected to improve the fitting quality and predictive accuracy of the model. Last but not least, the parameter E_Δ and the bandgap E_g are independent concepts in this context. Any attempt to use E_g obtained from experimental methods as an estimate for E_Δ to evaluate band nonparabolicity is strongly discouraged.

Note S3. Theoretical Bounds of the Nonparabolicity Factor (ξ) in Kane Model

Based on the Boltzmann transport theory, the Seebeck coefficient (S) in the Kane band model can be expressed as **Eq. S8**,

$$S = \frac{k_B}{q}\left[\frac{F^1_{1,-2}(\eta,\alpha)}{F^0_{1,-2}(\eta,\alpha)} - \eta\right] \tag{S8}$$

$$\sigma_s(\varepsilon,\alpha;n,m,k) = \varepsilon^n(\varepsilon+\alpha\varepsilon^2)^m[(1+2\alpha\varepsilon)^2+2]^{k/2} \tag{S9}$$

$$F^n_{m,k}(\eta,\alpha) = \int_0^{+\infty}\sigma_s(\varepsilon,\alpha;n,m,k)\cdot\left(-\frac{\partial f_0(\varepsilon;\eta)}{\partial\varepsilon}\right)d\varepsilon \tag{S10}$$

where k_B is the Boltzmann constant, q is the elementary charge, $\eta = E_F/k_BT$ is the reduced Fermi level, $\alpha = k_BT/E_\Delta$ is a dimensionless fitting parameter, $F^n_{m,k}(\eta, \alpha)$ is the generalized Fermi integral, and f_0 is the equilibrium Fermi-Dirac distribution. In the case of parabolic band (PB), one can simply take $\alpha = 0$. According to the definition in **Eq. 6** of body text, the nonparabolicity factor ξ can be expressed as **Eq. S8**,

$$\xi = \lim_{\eta\to-\infty}\frac{S^{PB}(\eta)-S(\eta)}{k_B/q} = \lim_{\eta\to-\infty}\frac{F^1_{1,-2}(\eta,0)}{F^0_{1,-2}(\eta,0)} - \frac{F^1_{1,-2}(\eta,\alpha)}{F^0_{1,-2}(\eta,\alpha)} \tag{S11}$$

As η approaches negative infinity, the Fermi-Dirac distribution f_0 can be equivalently represented by the Maxwell distribution, i.e.,

$$f_0(\varepsilon;\eta) = \frac{1}{1+e^{\varepsilon-\eta}} \to e^{\eta-\varepsilon} \tag{S12}$$

Taking the derivative with respect to ε yields **Eq. S13**,

$$-\frac{\partial f_0(\varepsilon;\eta)}{\partial\varepsilon} \to e^{\eta-\varepsilon} \tag{S13}$$

Consequently, $F^n_{m,k}(\eta, \alpha)$ can be expressed as **Eq. S14**,

$$F^n_{m,k}(\eta,\alpha) \to \int_0^{+\infty}\sigma_s(\varepsilon,\alpha;n,m,k)\cdot e^{\eta-\varepsilon}d\varepsilon = e^\eta\int_0^{+\infty}\sigma_s(\varepsilon,\alpha)\cdot e^{-\varepsilon}d\varepsilon \tag{S14}$$

In particular, when $\alpha = 0$ (corresponding to the PB model), $\sigma_s(\varepsilon, \alpha; n, m, k)$ simplifies to ε^{m+n}, and $F^n_{m,k}(\eta, \alpha)$ becomes mathematically equivalent to the *Gamma function*. Eventually, the former term in **Eq. S11** can be expressed as **Eq. S15**,

$$\lim_{\eta\to-\infty}\frac{F^1_{1,-2}(\eta,0)}{F^0_{1,-2}(\eta,0)} = \frac{e^\eta\int_0^{+\infty}\varepsilon^2\cdot e^{-\varepsilon}d\varepsilon}{e^\eta\int_0^{+\infty}\varepsilon\cdot e^{-\varepsilon}d\varepsilon} = 2 \tag{S15}$$

Clearly, when α equals 0, ξ reaches the lower bound of 0. As α approaches positive infinity, $\sigma_s(\varepsilon,$

α; n, m, k) can be expressed as **Eq. S16**,

$$\sigma_s(\varepsilon,\alpha;\ n,m,k) \to 2^k\alpha^{m+k}\varepsilon^{n+2m+k} \tag{S16}$$

Then,

$$\lim_{\eta\to-\infty}\frac{F_{1,-2}^{1}(\eta,\alpha)}{F_{1,-2}^{0}(\eta,\alpha)}=\frac{e^{\eta}\int_0^{+\infty}2^{-2}\alpha^{-1}\varepsilon\cdot e^{-\varepsilon}d\varepsilon}{e^{\eta}\int_0^{+\infty}2^{-2}\alpha^{-1}\cdot e^{-\varepsilon}d\varepsilon}=\frac{\int_0^{+\infty}\varepsilon\cdot e^{-\varepsilon}d\varepsilon}{\int_0^{+\infty}e^{-\varepsilon}d\varepsilon}=1 \tag{S17}$$

The upper bound of ξ can be expressed as **Eq. S18**,

$$\xi=\lim_{\eta\to-\infty}\frac{F_{1,-2}^{1}(\eta,0)}{F_{1,-2}^{0}(\eta,0)}-\lim_{\eta\to-\infty}\frac{F_{1,-2}^{1}(\eta,\alpha)}{F_{1,-2}^{0}(\eta,\alpha)}=2-1=1 \tag{S18}$$

Therefore, ξ varies within the range of 0 to 1.

Note S4. Electrical Transport Properties under Constrained E_Δ/m^*

In this work, the nonparabolicity factor (ξ) is introduced as an additional dimension to describe the energy dispersion relationship, complementing the intrinsic band effective mass (m^*) and enabling more accurate modeling of the electronic structure. In the main text, we examined the influence of ξ (or E_Δ) on thermoelectric transport properties, including electrical conductivity, Seebeck coefficient, power factor, and the *L*-*S* relationship, while keeping m^* constant, as depicted in **Figure S11(a)**. However, manipulating ξ in isolation while maintaining m^* unchanged is uncommon in practice. Here, we present a more feasible scenario where the band edge becomes sharper while deeper band regions remain nearly unchanged, as shown in **Figure S11(b)**. This phenomenon has been observed in Pb-doped SnS (Y. Xiao et al., J. Am. Chem. Soc. 142, 4051, 2020) and Dy-doped $CaTiO_3$ (Q. Jiang et al., Mater. Today Energy 44, 101655, 2024).

Within the Kane model framework, in deeper band regions, the E-$|k|$ relationship gradually transitions into a linear dependence governed by E_Δ/m^*. This can be derived by neglecting the first-order term on the left-hand side of **Eq. S19**:

$$E=\sqrt{\frac{E_\Delta}{2\,m^*}}\cdot|\hbar k| \tag{S19}$$

Thus, evaluating the influence of band nonparabolicity under the constrained condition of fixed E_Δ/m^* becomes particularly relevant. The main text establishes that, for constant weighted

mobility μ_{WT}, the peak power factor (PF_{max}) at the optimal doping concentration depends exclusively on ξ, as expressed in Equation S20:

$$PF_{max} = C \cdot (1-\xi)^2 \mu_{WT} \tag{S20}$$

where C is a material-independent physical constant (Zhu et al., Npj Comput. Mater. 7, 116, 2021). When m^* changes, μ_{WT} varies proportionally to $1/m^*$. By leveraging the proportional relationship between E_Δ and m^*, along with the correlation between E_Δ and ξ (**Figure 4(c)**), we can derive **Eq. S21**:

$$\mu_{WT} \propto \frac{1}{m^*} \propto \frac{1}{E_\Delta} \propto \frac{1}{\frac{2}{3}\left(\frac{1}{\xi}-1\right)} \propto \frac{\xi}{1-\xi} \tag{S21}$$

Substituting **Eq. S21** into **Eq. S21** yields:

$$PF_{max} \propto (1-\xi)^2 \mu_{WT} \propto (1-\xi)^2 \frac{\xi}{1-\xi} \propto \xi(1-\xi) \tag{S22}$$

Consequently, under the constrained condition of fixed E_Δ/m^*, PF_{max} exhibits non-monotonic behavior with ξ, reaching its maximum value at ξ = 0.5. This result suggests that moderate nonparabolicity can positively contribute to optimizing the power factor.

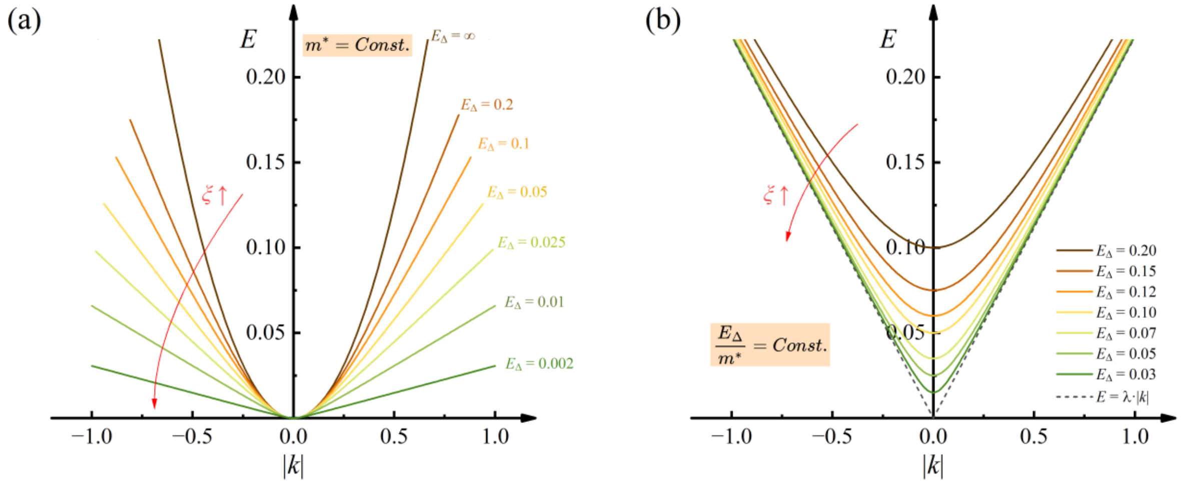

Figure S11. Illustrations of band nonparabolicity change scenarios. (a) Fixed band effective mass (m^*). (b) Fixed ratio of E_Δ/m^*. All quantities are in *atomic units* (E_h for energy, $\hbar/a_0$ for momentum, m_e for mass).

Note S5. Single Parabolic Band Effective Mass (m^*_{SPB})

Within the framework of the single parabolic band (SPB) model, the *S*-*n* relationship (Pisarenko plot) is solely determined by temperature (*T*) and the density-of-states effective mass (m^*_{DOS}). In experimental studies, an effective mass parameter (denoted as m^*_{SPB}) is commonly extracted from measured *S* and *n* data by arbitrarily applying the SPB model. Its anomalous variations can indicate underlying modifications in the band structure.

Here, we fix m^*_{DOS} = 1.0 m_e and T = 300 K, and investigate the influence of band nonparabolicity on the *S*-*n* relationship and m^*_{SPB} by varying the nonparabolicity factor ξ through adjustments to E_Δ (**Figures S12(a)** and **(b)**). Our results show that m^*_{SPB} initially plateaus above 1.0 m_e but begins to decrease at carrier concentrations $n > 10^{21}$ cm^{-3}. For thermoelectric materials, the optimal carrier concentrations are typically located in the range of $10^{19} \sim 10^{21}$ cm^{-3}. Therefore, it can be concluded that for the vast majority of experimental samples, band nonparabolicity leads to m^*_{SPB} being higher than the actual m^*_{DOS} of the material.

Quantitatively, when ξ reaches 0.09, the extracted m^*_{SPB} is approximately 1.12 m_e. When ξ reaches 0.33, m^*_{SPB} increases to approximately 1.5 m_e, corresponding to a 50% overestimation. In some extreme cases, when ξ reaches 0.75, m^*_{SPB} may be overestimated by up to four times the m^*_{DOS}, resulting in significant deviations that could heavily impact interpretation and design of thermoelectric materials.

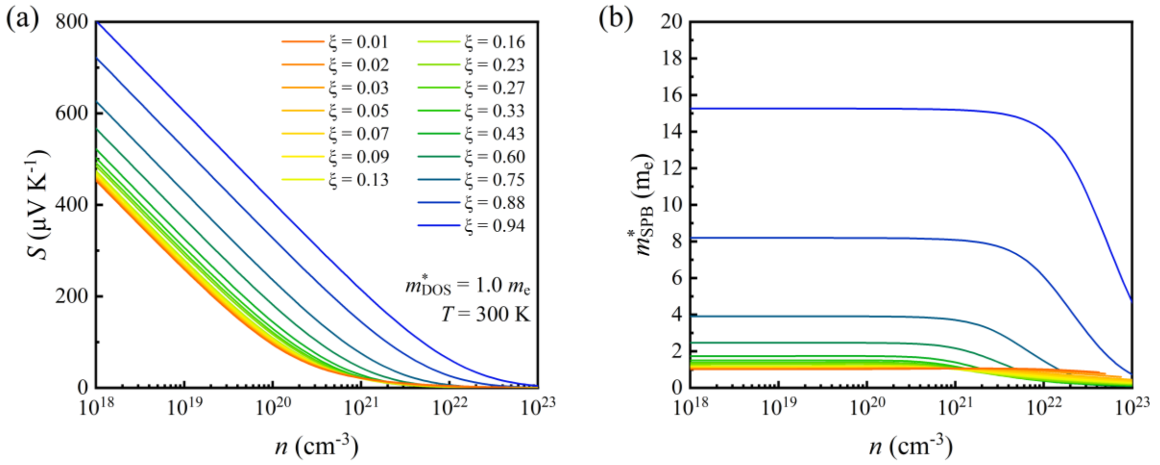

Figure S12. Influence of band nonparabolicity on the Pisarenko plot and effective mass estimation. **(a)** Calculated Pisarenko plot for varying nonparabolicity factors ξ, assuming m^*_{DOS} = 1.0 m_e and T = 300 K. **(b)** Effective mass extracted from Seebeck coefficient and carrier concentration within the framework of the single parabolic band (SPB) model.

Note S6. Band Nonparabolicity Beyond the Isotropic Kane Model

The isotropic Kane model can provide a reasonable approximation to the band dispersion near the band extrema over a wide range. This allows for more precise modeling of thermoelectric parameters with strong non-local characteristics. However, real band structures exhibit greater complexity, which challenges the applicability of the Kane model. In the main text, we introduced the concept of band nonparabolicity within the framework of the Kane model and proposed the nonparabolicity factor ξ. Here, we discuss its behavior under several complex band structures (as shown in **Figure S13**), including: (1) valley anisotropy, (2) multiple valleys, (3) subvalleys, (4) bipolar effects. The impact of these mechanisms on the power factor has been extensively discussed. Generally, valley anisotropy and band convergence are considered beneficial for achieving high power factors, whereas bipolar effects are detrimental. Here, we focus on the *L*-*S* relationship, which shows strong correlation with our proposed nonparabolicity factor ξ.

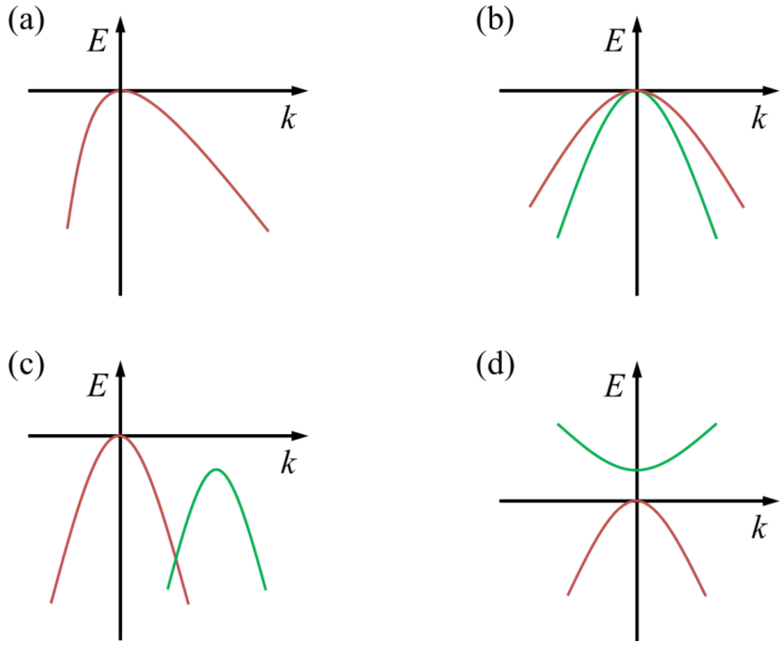

Figure S13. Complex band structures beyond the isotropic Kane model. **(a)** Valley anisotropy. **(b)** Multiple degenerate valleys. **(c)** Subvalleys near valence band maximum. **(d)** Narrow-gap bands with strong bipolar effects.

(1) Valley Anisotropy

The dispersion relation for the anisotropic Kane model can be expressed as **Eq. S23**:

$$E\left(1+\frac{E}{E_{\Delta}}\right)=\frac{\hbar^2 k_x^2}{2m_x^*}+\frac{\hbar^2 k_y^2}{2m_y^*}+\frac{\hbar^2 k_z^2}{2m_z^*} \tag{S23}$$

Based on Boltzmann transport theory (**Eqs. (1)-(4)** in the main text), it can be derived that $S_x = S_y = S_z$ and $L_x = L_y = L_z$, indicating that neither the Seebeck coefficient (S) nor the Lorenz number (L) is affected by valley anisotropy. This result arises from the fact that anisotropy simply scales the transport distribution function without altering its shape. Since both S and L are defined as ratios of weighted transport distribution functions, the influence of anisotropy is inherently eliminated.

(2) Multiple Valleys

Valley anisotropy has been demonstrated to have no effect on S and L; thus, the Cartesian directions are omitted here for simplicity. We begin by considering the case of two valleys, with their respective nonparabolicity factors and weighted mobility ratio denoted as ξ_1, ξ_2, and r. Based on Boltzmann transport theory, the total Seebeck coefficient can be expressed as **Eq. S24**:

$$S=\frac{S_1+rS_2}{1+r} \tag{S24}$$

Using the definition of the nonparabolicity factor in **Eq. (6)** from the main text, the nonparabolicity factor for the two-valley system can be derived as **Eq. S25**:

$$\xi=\frac{\xi_1+r\xi_2}{1+r} \tag{S25}$$

Combining **Eq. S24** and **Eq. S25**, the predicted Lorenz number (L_{pred}) for the material can be calculated using **Eqs. (8)-(11)** in the main text. Meanwhile, the real Lorenz number (L_{real}) derived from Boltzmann transport theory is given by **Eq. S26**:

$$L=\frac{L_1+rL_2}{1+r} \tag{S26}$$

When evaluating the prediction accuracy of the Lorenz number, four variables come into play: ξ_1, ξ_2, r, and the carrier concentration (n). To quantify the maximum error across doping levels, we define the relative deviation as **Eq. S27**:

$$\delta_{L,max}(\xi_1,\xi_2;r)=\max_{\frac{E_F}{k_BT}\in[-10,90]}\left|\frac{L_{pred}-L_{real}}{L_{real}}\right| \tag{S27}$$

Contour plots of $\delta_{L,max}(\xi_1, \xi_2)$ are presented in **Figure S14(a)-(e)** for r = 1, 2, 5, 10, and 20, respectively. Due to the interchangeable nature of ξ_1 and ξ_2, only cases with $r \geq 1$ are considered in the analysis. Moreover, the relationship between $\delta_{L,max}$ and $|\xi_1 - \xi_2|$ is summarized in **Figure**

S14(f), leading to two principal conclusions:

- **$\delta_{L,max}(\xi_1, \xi_2)$ is approximately proportional to $|\xi_1 - \xi_2|$**. This indicates that materials with multiple valleys exhibiting similar nonparabolicity characteristics enable more accurate predictions of the *L*-*S* relationship. Specifically, when $|\xi_1 - \xi_2| < 0.4$, the prediction error of L remains below 5%.

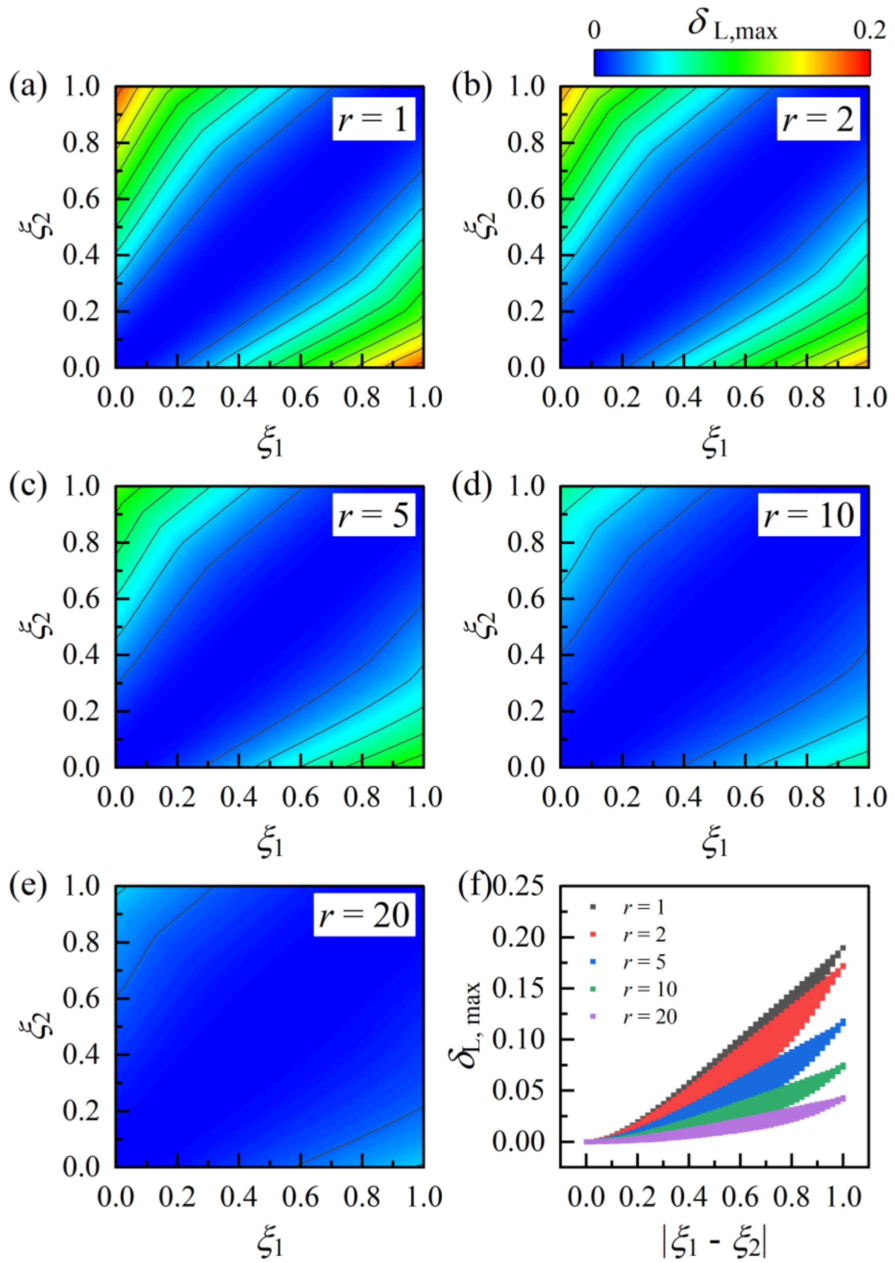

Figure S14. Lorenz number prediction accuracy for the two-valley system. **(a)-(e)** Contour plots of $\delta_{L,max}(\xi_1, \xi_2)$ for $r = 1$, 2, 5, 10, and 20, respectively. **(f)** Relationship between $\delta_{L,max}$ and $|\xi_1 - \xi_2|$.

- $\boldsymbol{\delta_{L,max}(\xi_1, \xi_2)}$ **decreases as r increases.** This reduction can be explained by the fact that both S and L are weighted averages governed by r, which reflects the relative contributions of mobility from different valleys. At large r values, transport properties are dominated by a single valley, effectively concealing the contribution of the other valley. Consequently, when r > 20, the prediction error of L stays below 5%, regardless of $|\xi_1 - \xi_2|$.

In summary, the accuracy of the proposed L-S analytical model in multi-valley materials is strongly dependent on the specific valley characteristics. The model demonstrates higher accuracy when the valleys exhibit matched nonparabolicity factors (i.e., $|\xi_1 - \xi_2|$ is small) but significantly different mobility ratios (i.e., r is large).

(3) Subvalleys

In addition to the band degeneracy protected by crystallographic symmetry, many high-performance thermoelectric materials feature subvalleys located near the valence band maximum or conduction band minimum, typically within a few k_BT. The thermoelectric performance model for such systems is analogous to the previously described "**(2) Multiple Valleys**" model. Specifically, the valley at the band edge is denoted as valley 1, while the inner subvalley is labeled as valley 2. The energy splitting between valley 1 and valley 2 is denoted as ΔE.

Based on the definition of the nonparabolicity factor ξ in **Eq. (6)** of the main text, the nonparabolicity factor for valley 2 can be expressed as $\xi_2' = \xi_2 - \Delta E/(k_BT)$, resulting in $|\xi_1 - \xi_2'| = |\xi_1 - \xi_2 + \Delta E/(k_BT)|$. According to previous conclusions, the maximum prediction error for the Lorenz number, $\delta_{L,max}$, is approximately proportional to $|\xi_1 - \xi_2'|$. This implies that large deviations in nonparabolicity, as introduced by subvalleys, may lead to significant prediction errors under certain conditions. Furthermore, $\delta_{L,max}$ is affected by the conductivity ratio, r, between carriers in the two valleys. Unlike degenerate valleys, the contribution of the subvalley conductivity evolves dynamically as the Fermi level E_F shifts from the band edge valley toward the subvalley, causing strong variability.

Taking SnTe as an example, r is defined as the conductivity ratio between the heavy band (σ_H) and the light band (σ_L). As shown in **Figure S15**, at the valence band maximum (E_{vbm}), r is approximately 10^{-6}, indicating that transport behavior is completely dominated by the light band. With increasing acceptor doping concentration, as the E_F shifts to -0.6 eV, r increases to 1. At this stage, the transport behavior has already become highly complex, exceeding the scope of our

proposed model.

In summary, for systems containing subvalleys, the proposed *L*-*S* analytical model is only applicable to intrinsic or lightly doped samples. Once subvalley contributions play a significant role in transport properties, this model is no longer recommended, and a more suitable model should be considered.

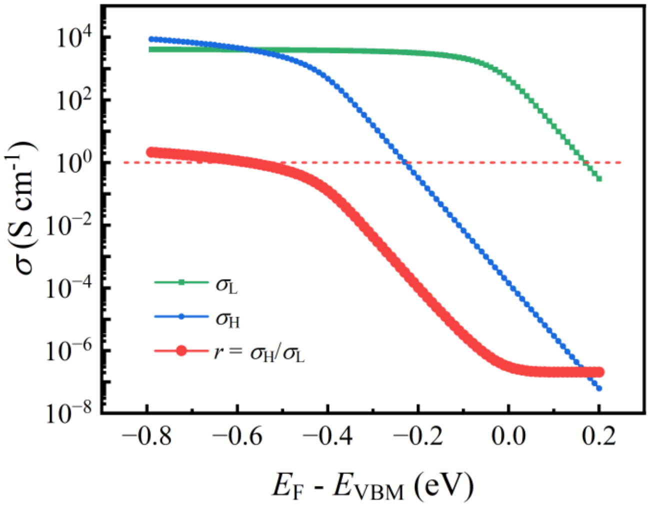

Figure S15. Band conductivities and their ratio in SnTe. Calculated at a fixed temperature of 300 K using parameters from the literature (M. Zhou et al., Phys. Chem. Chem. Phys., 16, 20741, 2014).

(4) Bipolar Effects

For narrow-bandgap semiconductors, electron-hole pairs can be significantly excited at high temperatures, leading to a remarkable increase in electronic thermal conductivity. In this context, the thermal conductivity originating from carrier drift is referred to as κ_{drift}, while the additional thermal conductivity introduced by intrinsic excitation is termed bipolar thermal conductivity, κ_{bip}. Further, the corresponding Lorenz numbers are defined as $L_{drift} = \kappa_{drift}/(\sigma T)$ and $L_{bip} = \kappa_{bip}/(\sigma T)$, where σ represents the total electrical conductivity and T is the temperature. Based on Boltzmann transport theory, L_{drift} and L_{bip} can be expressed as **Eq. S28** and **Eq. S29**:

$$L_{drift} = \frac{\sigma_e L_e + \sigma_h L_h}{\sigma_e + \sigma_h} \tag{S28}$$

$$L_{bip} = \frac{\sigma_e \sigma_h}{(\sigma_e + \sigma_h)^2}(S_h - S_e)^2 \tag{S29}$$

Here, the subscripts "e" and "h" represent electrons and holes, respectively. When intrinsic excitation occurs, the non-degenerate approximation becomes applicable, allowing the Fermi-Dirac distribution to be simplified into the Boltzmann distribution. Importantly, the proposed band nonparabolicity factor ξ is derived from the expression for the Seebeck coefficient under the non-degenerate approximation, as shown in **Eq. (6)** in the main text. By reverse derivation, incorporating the nonparabolicity factor modifies the Seebeck coefficient expression as:

$$S_e = -\frac{k_B}{q}\left(2 - \xi_e - \frac{E_{F,e}}{k_B T}\right) \quad \text{(S30)}$$

$$S_h = \frac{k_B}{q}\left(2 - \xi_h - \frac{E_{F,h}}{k_B T}\right) \quad \text{(S31)}$$

Considering $E_{F,e} + E_{F,h} = -E_g$, we obtain:

$$S_h - S_e = \frac{k_B}{q}\left(4 - \xi_e - \xi_h - \frac{E_{F,h}}{k_B T} - \frac{E_{F,e}}{k_B T}\right) = \frac{k_B}{q}\left(4 - \xi_e - \xi_h + \frac{E_g}{k_B T}\right) \quad \text{(S32)}$$

Thus, L_{bip} can be rewritten as **Eq. S33**:

$$L_{bip} = \left(\frac{k_B}{q}\right)^2 \frac{\sigma_e \sigma_h}{(\sigma_e + \sigma_h)^2}\left(4 - \xi_e - \xi_h + \frac{E_g}{k_B T}\right)^2 \quad \text{(S33)}$$

It can be observed that as ξ_e and ξ_h increase, the L_{bip} (and κ_{bip}) of the material decreases.

Moreover, both L_{drift} and L_{bip} depend on σ_e/σ_h, which requires experimental determination of the contributions of electrons and holes to the total conductivity. However, reliable methods for such separation are currently lacking and need further exploration. If relevant theories can be established, the proposed model will serve as an effective downstream tool for accurately estimating Lorenz numbers and bipolar thermal conductivity.

REFERENCES

1. Blöchl PE. Projector augmented-wave method. *Phys Rev B* 1994; **50**: 17953-79.
2. Kresse G, Furthmüller J. Efficient iterative schemes for *ab initio* total-energy calculations using a plane-wave basis set. *Phys Rev B* 1996; **54**: 11169-86.
3. Perdew JP, Burke K, Ernzerhof M. Generalized gradient approximation made simple. *Phys Rev Lett* 1996; **77**: 3865-8.
4. Wang Y, Perdew JP. Accurate and simple analytic representation of the electron-gas correlation energy. *Phys Rev B* 1992; **45**: 13244-9.
5. Fang T, Li X, Hu C *et al*. Complex band structures and lattice dynamics of Bi_2Te_3-based compounds and solid solutions. *Adv Funct Mater* 2019; **29**: 1900677.
6. Krukau AV, Vydrov OA, Izmaylov AF *et al*. Influence of the exchange screening parameter on the performance of screened hybrid functionals. *J Chem Phys* 2006; **125**: 224106.
7. Madsen GKH, Carrete J, Verstraete MJ. BoltzTraP2, a program for interpolating band structures and calculating semi-classical transport coefficients. *Comput Phys Commun* 2018; **231**: 140-5.
8. Ganose AM, Park J, Faghaninia A *et al*. Efficient calculation of carrier scattering rates from first principles. *Nat Commun* 2021; **12**: 2222.
9. Rogers LM. Valence band structure of SnTe. *J Phys D Appl Phys* 1968; **1**: 845-52.
10. Cahill DG, Watson SK, Pohl RO. Lower limit to the thermal conductivity of disordered crystals. *Phys Rev B* 1992; **46**: 6131-40.
